# An asymmetric structure of bacterial TrpRS supports the half-of-the-sites catalytic mechanism and facilitates antimicrobial screening

**DOI:** 10.1093/nar/gkad278

**Published:** 2023-04-18

**Authors:** Manli Xiang, Kaijiang Xia, Bingyi Chen, Zhiteng Luo, Ying Yu, Lili Jiang, Huihao Zhou

**Affiliations:** Guangdong Provincial Key Laboratory of Chiral Molecule and Drug Discovery and Research Center for Drug Discovery, School of Pharmaceutical Sciences, Sun Yat-sen University, Guangzhou, Guangdong510006, China; Guangdong Provincial Key Laboratory of Chiral Molecule and Drug Discovery and Research Center for Drug Discovery, School of Pharmaceutical Sciences, Sun Yat-sen University, Guangzhou, Guangdong510006, China; Guangdong Provincial Key Laboratory of Chiral Molecule and Drug Discovery and Research Center for Drug Discovery, School of Pharmaceutical Sciences, Sun Yat-sen University, Guangzhou, Guangdong510006, China; Guangdong Provincial Key Laboratory of Chiral Molecule and Drug Discovery and Research Center for Drug Discovery, School of Pharmaceutical Sciences, Sun Yat-sen University, Guangzhou, Guangdong510006, China; Guangdong Provincial Key Laboratory of Chiral Molecule and Drug Discovery and Research Center for Drug Discovery, School of Pharmaceutical Sciences, Sun Yat-sen University, Guangzhou, Guangdong510006, China; Guangdong Provincial Key Laboratory of Chiral Molecule and Drug Discovery and Research Center for Drug Discovery, School of Pharmaceutical Sciences, Sun Yat-sen University, Guangzhou, Guangdong510006, China; Guangdong Provincial Key Laboratory of Chiral Molecule and Drug Discovery and Research Center for Drug Discovery, School of Pharmaceutical Sciences, Sun Yat-sen University, Guangzhou, Guangdong510006, China

## Abstract

Tryptophanyl-tRNA synthetase (TrpRS) links tryptophan to tRNA^Trp^, thereby playing an indispensable role in protein translation. Unlike most class I aminoacyl-tRNA synthetases (AARSs), TrpRS functions as a homodimer. Herein, we captured an ‘open-closed’ asymmetric structure of *Escherichia coli* TrpRS (*Ec*TrpRS) with one active site occupied by a copurified intermediate product and the other remaining empty, providing structural evidence for the long-discussed half-of-the-sites reactivity of bacterial TrpRS. In contrast to its human counterpart, bacterial TrpRS may rely on this asymmetric conformation to functionally bind with substrate tRNA. As this asymmetric conformation is probably a dominant form of TrpRS purified from bacterial cells, we performed fragment screening against asymmetric *Ec*TrpRS to support antibacterial discovery. Nineteen fragment hits were identified, and 8 of them were successfully cocrystallized with *Ec*TrpRS. While a fragment named niraparib bound to the L-Trp binding site of the ‘open’ subunit, the other 7 fragments all bound to an unprecedented pocket at the interface between two TrpRS subunits. Binding of these fragments relies on residues specific to bacterial TrpRS, avoiding undesired interactions with human TrpRS. These findings improve our understanding of the catalytic mechanism of this important enzyme and will also facilitate the discovery of bacterial TrpRS inhibitors with therapeutic potential.

## Graphical Abstract

**Graphical Abstract:**
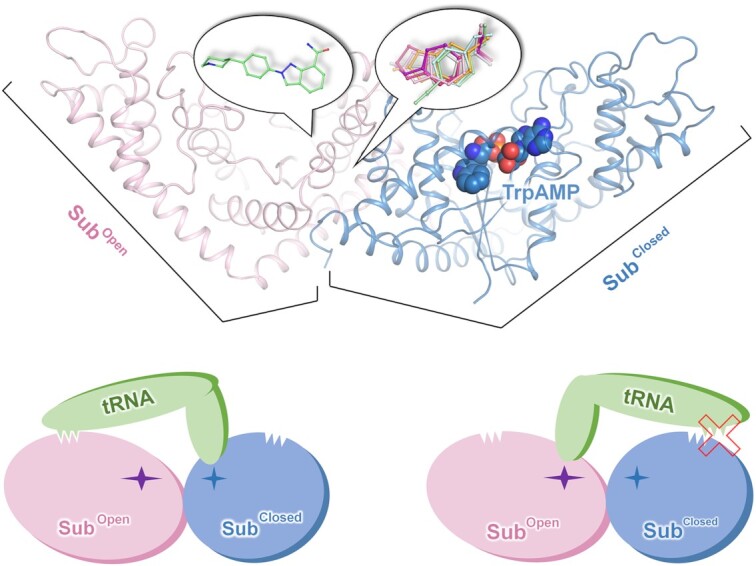
Crystal structures of Escherichia coli TrpRS reveal an “open-closed” asymmetric conformation which may be important for tRNA binding, and chemical fragments can bind to distinct pockets on the asymmetric TrpRS.

## INTRODUCTION

Aminoacyl-tRNA synthetases (AARSs) faithfully attach amino acids to their cognate tRNAs, and the ribosomes then use the produced aminoacyl-tRNAs as substrates to synthesize polypeptides based on the genetic information brought in mRNA (1). Proteins are generally synthesized with 20 proteinogenic amino acids; consequently, the AARS family mainly contains 20 members, each for a specific amino acid. All the AARS members are indispensable for protein translation, and their dysfunction disturbs cell viability and induces various diseases (2,3). On the other hand, inhibition of AARSs using small molecules is a promising strategy for treating multiple diseases, such as microbial infections (4,5), human cancers (6), fibrosis (7,8), and osteoporosis (9,10). To date, the AARS inhibitors mupirocin, tavaborole and halofuginone have been used in the clinic or as veterinary drugs (11–13), and more inhibitors are being evaluated at preclinical and clinical stages (14,15).

Tryptophanyl-tRNA synthetase (TrpRS) is the AARS member responsible for charging tRNA^Trp^ with L-tryptophan (L-Trp). TrpRS belongs to class I AARSs, and it is further grouped into subclass Ic together with tyrosyl-tRNA synthetase (TyrRS). Similar to other class I AARSs, TrpRS contains a Rossmann fold (RF) aminoacylation domain (AD) (16). Notably, TrpRSs from prokaryotes and eukaryotes show obvious sequence and structural differences at their active sites on AD and have developed distinct mechanisms to recognize the substrate L-Trp. The indole nitrogen of substrate L-Trp is coordinated by hydrogen bonding with a tyrosine residue located in a β-strand when binding to eukaryotic TrpRS but with an aspartate residue in an α-helix when binding to bacterial TrpRS (17). In contrast, the active site residues forming specific hydrogen-bonding interactions with amino acid substrates are usually conserved throughout evolution in other AARSs (17). Moreover, the charging of tRNA^Trp^ was also reported to be kingdom-specific that bacterial and eukaryotic TrpRSs can only catalyze their own tRNA^Trp^ substrates (18). Therefore, TrpRS is considered an attractive drug target for developing bacterial-selective inhibitors for fighting microbial infections (19). For example, two natural products, indolmycin and chuangxinmycin, have been shown to inhibit bacterial TrpRS at the nanomolar level with almost no undesired binding to human cytoplasmic TrpRS (*Hc*TrpRS) (20,21). However, unfortunately, both inhibitors failed to enter the clinical use due to insufficient permeability or narrow antibacterial spectrum, and inhibitors against bacterial TrpRS with new scaffolds and novel mechanisms are highly desired.

Different to most class I AARSs, TrpRSs in both prokaryotes and eukaryotes are homodimers (22). Previous studies have revealed a half-of-the-sites reaction mechanism for *Hc*TrpRS that once an intermediate product tryptophanyl adenylate (TrpAMP) is formed in one subunit, the second subunit can no longer efficiently produce TrpAMP (23). Despite the existence of obvious differences between human and bacterial TrpRSs at their active sites, the half-of-the-sites reactivity was also proposed for bacterial TrpRS because it was found to prefer to bind only one ATP at a time (24,25). Dozens of structures of TrpRS from various bacteria have been determined so far, but they all form symmetric conformations in which both subunits of bacterial TrpRS bind with the same ligands or both remain empty (25–28). Notably, bacterial TrpRS incubated with high concentrations of ATP or ATP + L-Trp was crystallized in a ‘closed-closed’ symmetric conformation with ATP or TrpAMP bound in both active sites (25,27), which somewhat conflicts with the proposed half-of-the-sites reactivity. There are not yet direct structural data to validate the half-of-the-sites catalytic mode for bacterial TrpRS, explain why bacterial TrpRS uses this catalytic mode, and, more importantly, explore the implications of this catalytic mode in drug discovery.

In this study, TrpRS from *Escherichia coli* (*Ec*TrpRS) was crystallized in an ‘open-closed’ asymmetric state with a copurified intermediate product TrpAMP bound to the ‘closed’ subunit. The structural modelling and binding assay revealed that only this ‘open-closed’ asymmetric *Ec*TrpRS is suitable for functionally binding of the substrate tRNA^Trp^, giving a biological relevance to using half of the sites in the catalysis of bacterial TrpRS. Considering that the asymmetric *Ec*TrpRS may represent the dominant conformation of TrpRS in bacterial cells, we used it as a template to screen chemical fragments. Nineteen fragment hits were identified, and the binding modes of 8 fragments to asymmetric *Ec*TrpRS were successfully clarified by cocrystal structures, providing valuable information for inhibitor design in the future.

## MATERIALS AND METHODS

### Protein preparation

The coding sequence of full-length *Ec*TrpRS (UniProt ID P00954) was amplified from the genomic DNA of the *E. coli* K12 strain and cloned into the pET20b (+) plasmid (Novagen) with a hexahistidine tag at its C-terminus. BL21 (DE3) cells transformed with the *Ec*TrpRS-pET20b (+) plasmid were grown in Luria-Bertani (LB) medium supplemented with 100 mg/l ampicillin at 37°C until the OD600 = 0.6–0.8. Then, 0.15 mM isopropyl-β-d-thiogalactoside (IPTG) was added, and the bacterial cells were further cultured at 16°C for 12 h. Cells were harvested using centrifugation, resuspended in wash buffer (400 mM NaCl, 50 mM Tris–HCl pH 8.0, 5% glycerol and 20 mM imidazole), and broken by sonication. The cell lysate was loaded onto a Ni-NTA column (Qiagen) pre-equilibrated with wash buffer. The impurities were washed away with 20 columns of wash buffer, and then the target protein was eluted using elution buffer (400 mM NaCl, 50 mM Tris–HCl pH 8.0, 5% glycerol, and 100 mM imidazole). The target protein was concentrated and further purified using HiLoad 16/60 Superdex 200 pg (GE healthcare) with running buffer (200 mM NaCl, 20 mM Tris–HCl pH 8.0, and 5% glycerol). Protein purity was confirmed by sodium dodecyl sulfate–polyacrylamide gel electrophoresis (SDS−PAGE). The target protein was finally concentrated to 60 mg/ml in storage buffer (50 mM NaCl, 2 mM Tris–HCl pH 8.0, and 5% glycerol) and stored at -80°C before use. *Staphylococcus aureus* TrpRS (*SaTrpRS*, residues 1–329) and N-terminal truncated *Hc*TrpRS (residues 48–471) were expressed and purified similarly to *Ec*TrpRS.

### Crystallography

*Ec*TrpRS was crystallized using the sitting-drop vapor-diffusion method. Each crystallization drop consisted of 1 μl *Ec*TrpRS protein (30 mg/ml) and 1 μl reservoir solution (0.16 M ammonium sulfate, 0.1 M HEPES pH 7.5, 25% PEG3350) and was equilibrated against 100 μl of reservoir solution at 18°C for 1–3 days to allow crystals to grow. Large crystals were immersed in cryoprotectant solution (reservoir solution supplemented with 20% ethylene glycol) for a few seconds and then flash-frozen in liquid nitrogen. For growing cocrystals of the *Ec*TrpRS·fragment complexes, the selected fragments (2–5 mM) were mixed with *Ec*TrpRS (30 mg/ml) and incubated on ice for 30 min before setting up crystallization drops.

The diffraction data were collected at 100 K at the BL19U1 beamline of the National Facility for Protein Sciences Shanghai (NFPSS), the BL02U1 beamline of the Shanghai Synchrotron Radiation Facility (SSRF) and an in-house Xcalibur Nova single-crystal diffractometer, and processed using XDS (29) and CrysAlisPro software (Agilent Technologies UK Ltd). Structures were solved using the program Molrep (30) by the molecular replacement method with the ‘open-open’ *Ec*TrpRS structure (PDB code 5V0I) as the search model. Coot (31) and Refmac5 (32) were used iteratively to refine the structural models. The stereochemical quality of the final models was assessed using MolProbity (33). The statistics of data collection and structural refinements are listed in Supplementary Table S1. The coordinates and structural factors of the structures described in this paper have been deposited in the Protein Data Bank (PDB) under the accession codes 8I1W (TrpAMP), 8I4I (TrpAMP and L-Trp), 8I27 (TrpAMP and M1-67), 8I2A (TrpAMP and M1-109), 8I1Z (TrpAMP and M1-158), 8I2C (TrpAMP and M2-54), 8I2J (TrpAMP and M2-140), 8I1Y (TrpAMP and M3-108), 8I2L (TrpAMP and chlorzoxazone), and 8I2M (TrpAMP and niraparib).

### Fluorescence-based thermal shift assay

Ligand binding usually stabilizes a protein during its thermal denaturation process, resulting in a positive shift of the protein melting temperature compared to the apo protein (34). The fluorescence-based thermal shift assay (TSA) was employed for fragment screening against asymmetric *Ec*TrpRS bound with a molecule of TrpAMP. The 20 μl reactions, consisting of 150 mM NaCl, 100 mM MES pH 6.5, 2 μg *Ec*TrpRS, 4 × SYPRO orange fluorescence dye (Sigma-Aldrich) and 1 mM of one of the tested fragments, were prepared in the 96-well plates (Life Technologies) on ice. The reaction without adding any fragment was used as the blank control. The plates were incubated at 25°C for 10 min and then heated from 25°C to 95°C at a rate of 1°C/min. The fluorescence intensity was recorded every 30 s using a StepOne Plus™ RT-PCR equipment. The melting temperatures (*T*_m_) of *Ec*TrpRS with and without fragments were calculated using StepOne™ software v2.3. The average *T*_m_ values of triplicate assays were used. A fragment was considered as a positive hit when the Δ*T*_m_ between *Ec*TrpRS with and without adding this fragment was greater than 1°C. Furthermore, a parallel fragment screening against the ‘open-open’ *Ec*TrpRS (prepared by dialysis of the ‘open-closed’ asymmetric *Ec*TrpRS) was performed as a control.

### Preparation of tRNA^Trp^

For the ATP consumption assay, *E. coli* tRNA^Trp^ was prepared by overexpression in *E. coli*. The DNA sequence encoding *E. coli* tRNA^Trp^ (CCA) (tRNAdb ID tdbD00011300) was synthesized and inserted into the pET20b (+) vector between the T7 promoter and T7 terminator through homologous recombination. The transformed *E. coli* BL21 (DE3) cells were cultured in LB medium until the OD600 reached ∼0.6, and 1 mM IPTG was added to induce the overexpression of tRNA^Trp^ (CCA) at 30°C for 16 h. The tRNA^Trp^ (CCA) was extracted from cell pellets using RNAiso Plus (TakaRa) and chloroform, and precipitated from aqueous fractions using isopropanol. The tRNA pellet after centrifugation was redissolved using a buffer consisting of 20 mM Tris–HCl pH 8.0 and 1 mM EDTA, and loaded onto a HiTrap Q XL (GE healthcare). The column was eluted with a linear gradient of NaCl (0–1.0 M) supplemented with 20 mM Tris–HCl pH 8.0 and 10 mM MgCl_2_. The fractions containing tRNA were concentrated to 10 mg/ml and stored at –80°C in a buffer consisting of 10 mM HEPES pH 7.5 and 10 mM MgCl_2_.

Transfer of L-Trp from the intermediate product TrpAMP to the 3’ end of tRNA^Trp^ will trigger a state transition of *Ec*TrpRS. Thus, an A76-truncated tRNA^Trp^ was prepared with *in vitro* T7 polymerase transcription and used in the EMSA to test the binding of tRNA^Trp^ to *Ec*TrpRS at different states. The DNA template of *E. coli* tRNA^Trp(ΔA76)^ (CCA) with a T7 promoter fused at the 5’ end was synthesized using PCR with primer 1 (5'-**TAATACGACTCACTATA**AGGGGCGTAGTTCAATTGGTAGAGCACCGGTCTCCAAAACC-3') and primer 2 (5'-GGCAGGGGCGGAGAGACTCGAACTCCCAACACCCGGTTTTGGAGACCGGTGCT-3'). These two primers covered the full sequence of *E. coli* tRNA^Trp(ΔA76)^ (CCA) and partially overlapped with each other (underlined nucleotides), and primer 1 also contained a T7 promoter sequence (nucleotides in bold). The PCR product was further amplified by the second round of PCR with primer 3 (5'-TAATACGACTCACTATAAGGGGCGTAG-3') and primer4 (5'- *GG*CAGGGGCGGAGAGACTCGA-3'), and the product was then used as the DNA template for the *in vitro* T7 transcription assay without additional purification. The last two nucleotides at the 5’ terminus of primer 4 (nucleotides in italics) were methylated at their 2’-hydroxyl groups to reduce nontemplated nucleotide addition by the T7 RNA polymerase (35). The *in vitro* transcription reaction mixture contained 200 mM Tris–HCl pH 8.0, 20 mM MgCl_2_, 2 mM spermidine, 10 mM DTT, 4 mM ATP, 4 mM UTP, 4 mM CTP, 4 mM GTP, 50 μg/ml template DNA, and 1 μM T7 polymerase. After incubation at 37°C for 3–4 h, the transcripts were denatured at 95°C for 5 min. The tRNA transcripts were purified using 10% PAGE supplemented with 8 M urea. The tRNA band was cut, and tRNA was extracted from the gel using 0.5 M ammonium acetate and precipitated by ethanol. The tRNA was redissolved in a buffer consisting of 20 mM Tris–HCl pH 8.0 and 1 mM EDTA, heated at 65 °C for 5 min, and then refolded by slowly cooling to room temperature after the addition of 10 mM MgCl_2_. The refolded tRNA was concentrated to 10 mg/ml, aliquoted, and stored at –80°C for future use.

### ATP consumption assay

The inhibition of the aminoacylation activity of *Ec*TrpRS by different ligands was measured using an ATP consumption assay (36). *Ec*TrpRS was incubated with 1 mM of each ligand for 20 min on ice, and then substrates were added to initiate the reactions. The 10 μl reactions, consisting of 50 nM *Ec*TrpRS, 4 μM ATP, 10 μM L-Trp, 0.4 mg/ml *E. coli* tRNA^Trp^ (CCA) (prepared by overexpression in *E. coli*), 30 mM HEPES pH 7.5, 150 mM NaCl, 30 mM KCl, 40 mM MgCl_2_, 1 mM DTT and 0.1% BSA, were incubated in a 384-well microplate at 37°C for 10 min. Then, 10 μl of Kinase-Glo® Reagent (Promega) was added to each well to measure the amount of remaining ATP. After 10 min of incubation, the luminescence (*L*) was read on a FlexStation 3 multimode microplate reader (Molecular Devices). The luminescence intensity of the sample without any inhibitor was used as L_min_, and the luminescence intensity of the sample without *Ec*TrpRS was used as *L*_max_. The inhibitory rate of a ligand against *Ec*TrpRS was calculated as inhibitory rate = (*L* – *L*_min_)/(*L*_max_ – *L*_min_) × 100%. The reactions were each repeated three times. For niraparib, its inhibitory rates at different concentrations (31.25, 62.5, 125, 250, 500 and 1000 μM) were measured, and then its IC_50_ was calculated by fitting a does-response curve using GraphPad Prism 8 software.

### Isothermal titration calorimetry (ITC) assay

The binding affinities of ligands to *Ec*TrpRS were measured by using a MicroCal VP-ITC microcalorimeter (MicroCal). The proteins and ligands were prepared in PBS buffer (137 mM NaCl, 2.7 mM KCl, 8 mM Na_2_HPO_4_, and 2 mM KH_2_PO_4_). The titration assays were performed at 15°C with 0.4 μl for the first injection and 2 μl each for the next 19 injections, and the interval between two injections was 120 s. The disassociation constants (*K_d_*) were determined by fitting the calorimetric data to the one-site binding model using MicroCal PEAQ-ITC analysis software, and the errors of *K*_d_ values represented the curving fitting errors. All of the above ITC experiments were repeated at least twice, and titrations of ligands to *Hc*TrpRS and *Sa*TrpRS were also performed for comparison.

### Construction of the *Ec*TrpRS·tRNA^Trp^ complex models

The docking of tRNA^Trp^ to asymmetric *Ec*TrpRS was based on a cocrystal structure of *Hc*TrpRS bound with two tRNA^Trp^ molecules (PDB code 2DR2) (18). To build the *Ec*TrpRS·tRNA^Trp^ binding model I, one tRNA^Trp^ molecule was removed from the *Hc*TrpRS·tRNA^Trp^ complex, and the remaining *Hc*TrpRS·tRNA^Trp^ complex was then overlaid on *Ec*TrpRS by aligning the AD of its subunit bound with the tRNA^Trp^ acceptor stem with the AD of the sub^Closed^ of *Ec*TrpRS. Two α-helices (Y100-R106 and R158-A166) on the AD of *Ec*TrpRS recognized the acceptor stem of tRNA^Trp^ as their corresponding helices did in the *Hc*TrpRS·tRNA^Trp^ complex, and the CCA end of tRNA^Trp^ was shown to approach TrpAMP in the active site of sub^Closed^ to receive the L-Trp. We kept the interactions between the tRNA^Trp^ acceptor stem and sub^Closed^ and slightly rotated tRNA^Trp^ to move its anticodon arm towards the CTD of sub^Open^ of *Ec*TrpRS. To build the *Ec*TrpRS·tRNA^Trp^ binding model II, the *Hc*TrpRS·tRNA^Trp^ complex was first overlaid on the sub^Open^ of *Ec*TrpRS, and then the anticodon arm of tRNA^Trp^ was rotated towards the CTD of sub^Closed^ of *Ec*TrpRS. However, in binding model II, the anticodon binding site of the sub^Closed^ of *Ec*TrpRS was unable to reach the tRNA^Trp^ anticodon triplets because the distance between the anticodon binding site of sub^Closed^ and acceptor stem binding site of sub^Open^ of *Ec*TrpRS is shorter than the distance between their corresponding binding elements on tRNA^Trp^, suggesting that tRNA^Trp^ cannot functionally bind to *Ec*TrpRS in binding model II.

### Electrophoretic mobility shift assay

An electrophoretic mobility shift assay (EMSA) was performed to explore the differences in the binding of tRNA^Trp^ to *Ec*TrpRS in different states. As shown in the crystal structure, the two subunits of purified *Ec*TrpRS mainly adopted the ‘open-closed’ asymmetric conformation. The ‘open-open’ *Ec*TrpRS was prepared via dialysis. Briefly, the purified ‘open-closed’ asymmetric *Ec*TrpRS was diluted to 1 mg/ml, loaded onto a dialysis bag (cut-off molecular weight of 3000 Da), and dialyzed against 1 L dialysis buffer (300 mM NaCl, 50 mM Tris–HCl pH 9.0, 5% glycerol and 30 mM L-Trp) twice at 8°C to remove the copurified TrpAMP. The ‘closed-closed’ *Ec*TrpRS was prepared by incubating *Ec*TrpRS with 2 mM ATP and 2 mM L-Trp on ice for 30 min as described in the literature (27).

*Ec*TrpRS at different states was diluted to 10 μM, 20 μM, 40 μM and 80 μM in the buffer consisting of 30 mM sodium cacodylate pH 6.5, 40 mM MgCl_2_, 150 mM NaCl, 30 mM KCl, 2 mM DTT and 10% glycerol, and incubated with tRNA^Trp(ΔA76)^ (5 μM) at 4°C for 30 min. The mixtures were loaded onto a 5% native polyacrylamide gel and electrophoresed at a voltage of 80 V for 2 h on ice. The gel was dyed with GelRed and then with Coomassie brilliant blue R-250.

## RESULTS

### An ‘open-closed’ asymmetric structure supports the long-discussed half-of-the-sites reactivity of bacterial TrpRS

**Figure 1.**
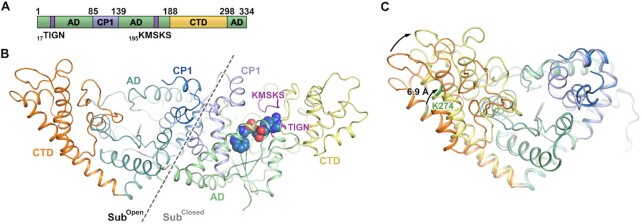
The ‘open-closed’ asymmetric structure of *Ec*TrpRS. (**A**) The domain organization of *Ec*TrpRS. (**B**) The overall structure of homodimeric *Ec*TrpRS in an asymmetric conformation with a molecule of the intermediate product TrpAMP bound at the active site cavity of the closed subunit (sub^Closed^). *Ec*TrpRS is presented as a cartoon model and colored according to (A), and TrpAMP is presented as a sphere. (**C**) Superposition of sub^Open^ and sub^Closed^ by aligning their ADs.

In this study, a crystal structure of full-length *Ec*TrpRS·TrpAMP was solved at 1.80 Å resolution with *R*/*R*_free_ = 18.6%/22.2% (Supplementary Table S1). The asymmetric unit contains two *Ec*TrpRS subunits that form a functional homodimer. Each *Ec*TrpRS subunit consists of a Rossmann fold aminoacylation domain (AD, residues 1–85, 140–183 and 299–334), a connecting polypeptide 1 domain [CP1, residues 86–139; CP1 was combined with AD in some previous studies (37)] and a C-terminal α-helical domain (CTD, residues 184–298) (Figure 1A).

Intriguingly, two subunits of this *Ec*TrpRS dimer adopt different conformations. A copurified tryptophanyl adenylate (TrpAMP), the intermediate product of the two-step tRNA tryptophanylation reaction, was observed to bind to one of the two active sites of *Ec*TrpRS (Figure 1B and Supplementary Figure S1A). The crystal structure of *Ec*TrpRS in complex with L-Trp and AMP has been determined (PDB code 5V0I) (Supplementary Figure S1B). The L-Trp moiety of TrpAMP interacts with *Ec*TrpRS similarly to the substrate L-Trp, except that the side chain of Tyr128 rotates downwards (∼5.3 Å for the phenolic hydroxyl) to interact with the α-amino group of the L-Trp moiety of TrpAMP (Supplementary Figure S1C). Compared with the AMP molecule, the AMP portion of TrpAMP moves approximately 6.0 Å to attach to the L-Trp moiety, and the class I AARS signature motifs TIGN (equal to HIGH in other class I AARSs) and KMSKS of *Ec*TrpRS shift together with the AMP part of TrpAMP towards the active site (Supplementary Figure S2A). The shift of these two motifs couples with an inwards rotation of the CTD around a short hinge linker located at the beginning of the CTD (residues 184–188) (37), which makes the TrpAMP-bound subunit a closed active site cavity and a more compact overall conformation (named sub^Closed^) compared with *Ec*TrpRS bound with L-Trp and AMP (Supplementary Figure S2A). In contrast, the active site cavity of the other subunit of *Ec*TrpRS dimer remains empty, and this empty subunit displays an open conformation (named sub^Open^) similar to that when L-Trp and AMP bind (Supplementary Figure S2B). Aligning sub^Open^ and sub^Closed^ by superimposing their ADs shows that the anticodon binding site at the distal end of the CTD of sub^Closed^ moves ∼6.9 Å towards the active site compared with that of sub^Open^ (Figure 1C). Thus, this *Ec*TrpRS dimer is in an ‘open-closed’ asymmetric conformation unique to all the reported structures of bacterial TrpRS.

**Figure 2.**
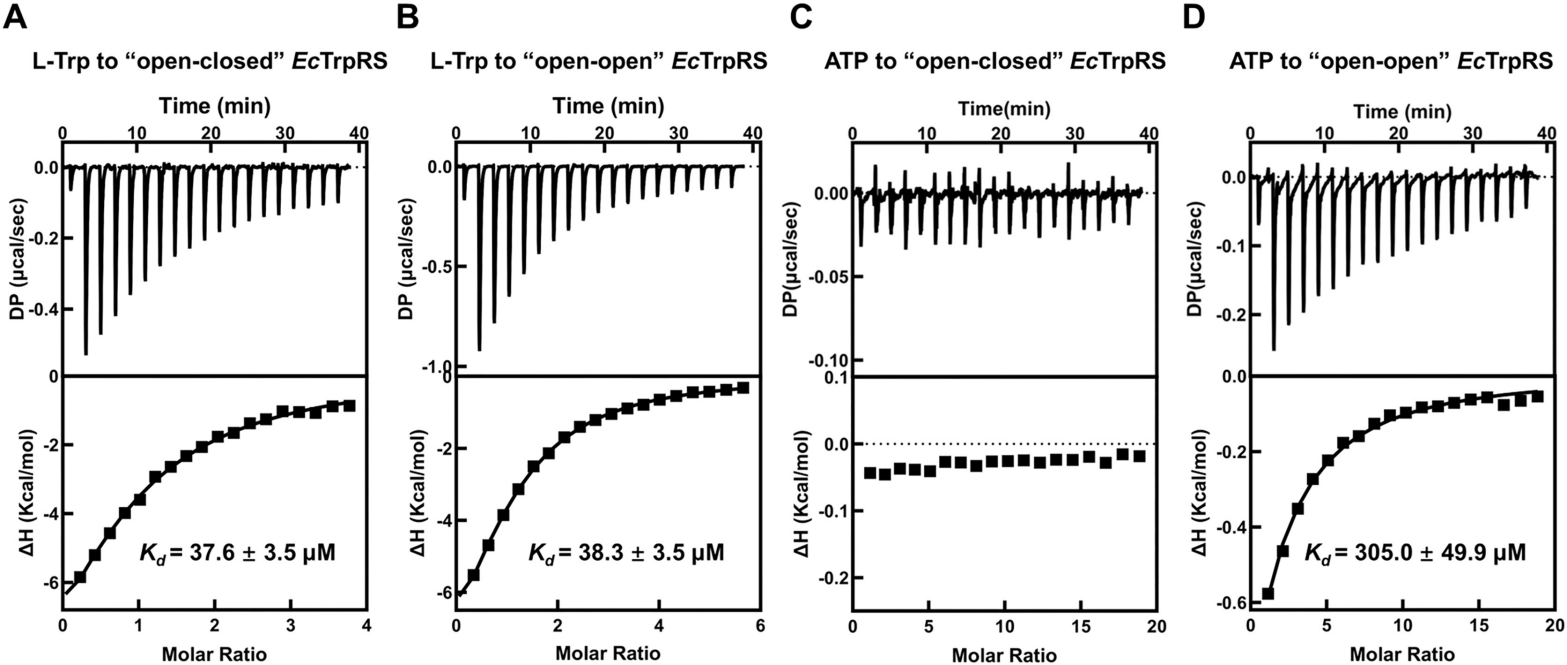
Binding affinities of substrates L-Trp and ATP to *Ec*TrpRS in asymmetric and apo states as measured by isothermal titration calorimetry. (**A**, **B**) ITC measurements of L-Trp to ‘open-closed’ asymmetric *Ec*TrpRS and ‘open-open’ apo *Ec*TrpRS showed similar binding affinities, revealing that occupying one active site by the intermediate product TrpAMP does not affect the binding of L-Trp to the other active site. (**C**, **D**) ITC measurements of ATP to ‘open-closed’ *Ec*TrpRS and ‘open-open’ *Ec*TrpRS showed that once an active site binds with a TrpAMP, the second active site could not recruit ATP efficiently.

Production of TrpAMP requires the simultaneous binding of both substrates L-Trp and ATP to the active site cavity. We tried to supplement the asymmetric *Ec*TrpRS with excessive substrates (either 2 mM L-Trp or 2 mM ATP) during crystallization. Only L-Trp, but not ATP, was successfully cocrystallized with the asymmetric *Ec*TrpRS in the active site cavity of its sub^Open^ (Supplementary Figure S3 and Supplementary Table S1). We also employed isothermal titration calorimetry (ITC) assays to compare the differences in affinities of both substrates between binding to ‘open-closed’ asymmetric *Ec*TrpRS and binding to apo *Ec*TrpRS (prepared by dialysis). The binding of TrpAMP in one subunit of *Ec*TrpRS caused little change in the affinity of L-Trp to the rest active site in the other subunit, but it significantly decreased the affinity of ATP compared with apo *Ec*TrpRS (Figure 2A–D). These results suggested that *Ec*TrpRS formed this ‘open-closed’ asymmetric conformation because it is unfavorable to bind with an additional ATP, which is consistent with the previous findings that *Bacillus stearothermophilus* TrpRS prefers to bind only one substrate ATP at a time (25). However, it is unclear how the two active sites of the bacterial TrpRS dimer communicate with each other in regard to ATP binding. A short CP1 sequence (residues Gln109 to Ser114) is disordered in sub^Open^ but forms an α-helix in TrpAMP-bound sub^Closed^ (Figure 1B). This short α-helix could contribute to stabilizing the conformation of the KMSKS loop, a loop important for ATP binding (Supplementary Figure S4). This short CP1 sequence in two TrpRS subunits may affect each other by involving in a unified large hydrophobic interaction network (Supplementary Figure S4), which provides a possible way to bridge two ATP binding sites of the TrpRS dimer.

### The asymmetric conformation of *Ec*TrpRS is needed for the functional docking of substrate tRNA^Trp^

**Figure 3.**
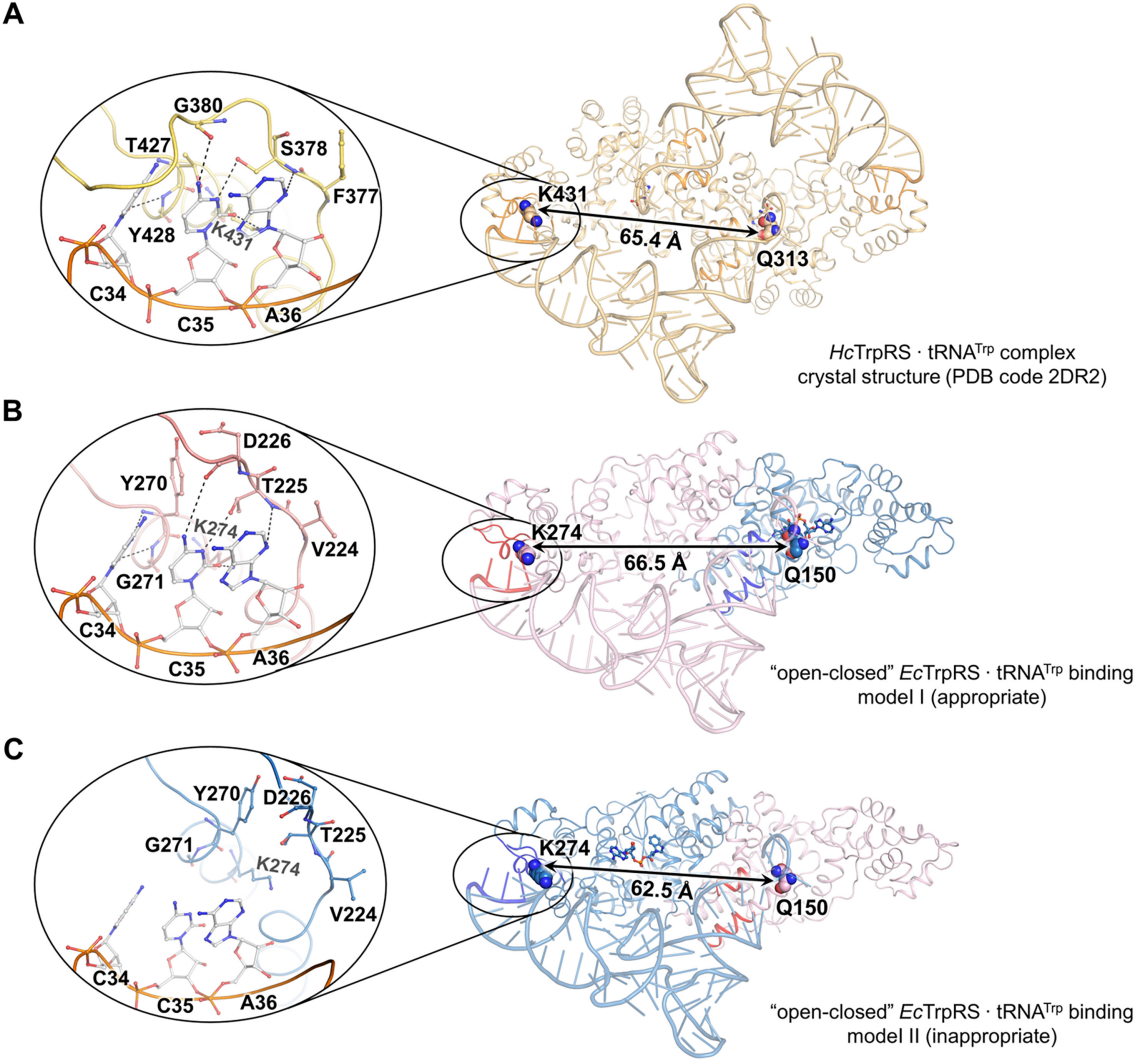
The binding mode of *Ec*tRNA^Trp^ asks TrpRS to apply the half-of-the-sites reactivity. (**A**) Crystal structure of the *Hc*TrpRS·tRNA^Trp^·Trp complex (PDB code 2DR2). (**B**, **C**) Docking of tRNA^Trp^ to ‘open-closed’ *Ec*TrpRS in two opposite ways·tRNA^Trp^·TrpAMP complex. The distance between the anticodon binding site of sub^Closed^ and acceptor stem binding site of sub^Open^ of *Ec*TrpRS is shorter than the corresponding distance observed in cocrystal structure of *Hc*TrpRS·tRNA^Trp^ complex, suggesting that tRNA^Trp^ cannot functionally bind to *Ec*TrpRS in this way. In the enlarged views of tRNA anticodon binding sites, the tRNA anticodon binding sites are shown as orange (A), pink (B) and skyblue (C) sticks, respectively, while the anticodon nucleotides as white sticks.

The kingdom-specific recognition of bacterial and eukaryotic tRNA^Trp^ molecules by their corresponding TrpRSs could be reversed by just switching their discriminator nucleotide N73 on tRNA^Trp^ molecules or the N73 interacting residues on their corresponding TrpRSs (38). Thus, the overall structure and the major interactions with TrpRS (for both the anticodon and acceptor stem) should be similar between bacterial and eukaryotic tRNA^Trp^. The complex structures of *Hc*TrpRS bound with tRNA^Trp^ have been solved (PDB codes: 2DR2 and 2AZX), revealing a cross-subunit tRNA binding mode typically observed in class II AARSs (18,39). In this binding mode, the acceptor stem of tRNA^Trp^ is approached through its major groove side by two α-helices on the AD of one *Hc*TrpRS subunit, and the anticodon of tRNA^Trp^ is recognized by the anticodon binding site located at the distal end of the other subunit (Figure 3A) (18,39).

We modeled tRNA^Trp^ to asymmetric *Ec*TrpRS based on the cocrystal structure of the *Hc*TrpRS·tRNA^Trp^ complex. We found that the distance between the acceptor stem binding helices of sub^Closed^ and the anticodon binding site of sub^Open^ perfectly matches the distance between their corresponding interacting elements on tRNA^Trp^ (Figure 3B), suggesting that tRNA^Trp^ is able to bind to *Ec*TrpRS in this way. In contrast, because of the closed conformation induced by TrpAMP, the distance between the acceptor stem binding helices and anticodon binding site of sub^Closed^ is approximately 4.0 Å shorter [the distance between the Cα atoms of K274 and Q150, two residues corresponding to K431 and Q313 in *Hc*TrpRS that play central roles in recognizing the anticodon and acceptor stem of tRNA^Trp^, respectively (18)] than that of sub^Open^ (Supplementary Figure S5). When the acceptor stem of tRNA^Trp^ was docked onto the AD of sub^Open^, its anticodon, particularly nucleotide C34, stretched to outside of the anticodon binding site of sub^Closed^, suggesting that tRNA^Trp^ could not functionally bind to *Ec*TrpRS in this way (Figure 3C). For similar reasons, two tRNA^Trp^ could be modeled onto ‘open-open’ *Ec*TrpRS, but no tRNA^Trp^ could be modeled onto ‘closed-closed’ *Ec*TrpRS (Supplementary Figure S5).

**Figure 4.**
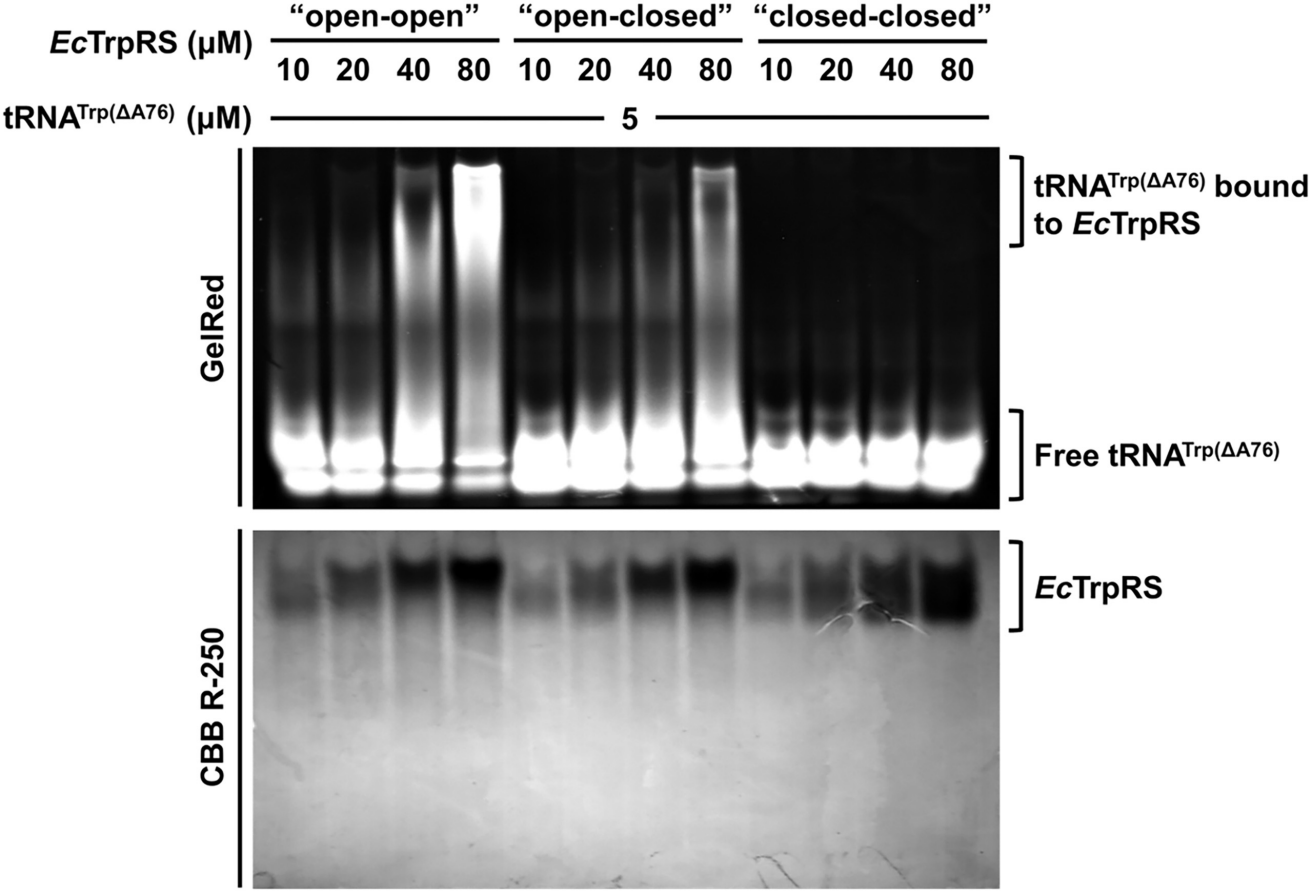
Electrophoretic mobility shifts of *Ec*tRNA^Trp(ΔA76)^ with *Ec*TrpRS in different conformations. tRNA^Trp(ΔA76)^ was shifted in a does-dependent manner by the ‘open-open’ and ‘open-closed’ *Ec*TrpRSs but not by the ‘closed-closed’ *Ec*TrpRS, suggesting that capturing a substrate *Ec*tRNA^Trp^ by *Ec*TrpRS requires at least one subunit of *Ec*TrpRS to adopt an open conformation, which agrees with the half-of-the-sites reactivity. The gel was dyed with GelRed and then with Coomassie brilliant blue R-250.

Notably, the potential flexibility and conformation changes of tRNA^Trp^, which are hard to predict, were not taken into account for the above docking model. Thus, to examine the binding models, we performed an electrophoretic mobility shift assay (EMSA) to detect the binding of tRNA^Trp^ to *Ec*TrpRS in different states. While the transfer of L-Trp from TrpAMP to tRNA^Trp^ will change the state of *Ec*TrpRS, the *E. coli* tRNA^Trp^ without A76 (*Ec*tRNA^Trp(ΔA76)^) was used in the experiments. The results showed that tRNA^Trp(ΔA76)^ was shifted in a does-dependent manner by the apo (‘open-open’ conformation; prepared by dialysis) and the asymmetric (‘open-closed’ conformation) *Ec*TrpRS. However, the shift of tRNA^Trp(ΔA76)^ by *Ec*TrpRS bound with two TrpAMPs [prepared by incubating *Ec*TrpRS with high concentrations of ATP and L-Trp as described (27)] was not observed (Figure 4), indicating that the ‘closed-closed’ conformation of *Ec*TrpRS is unfavorable for tRNA binding and that the binding of tRNA^Trp^ to *Ec*TrpRS requires at least one subunit to adopt the open conformation. Thus, interestingly, although the half-of-the-sites reactivity of bacterial TrpRS is apparently due to the unfavourable binding of the second ATP (the first step of the aminoacylation reaction catalyzed by TrpRS), our structural analysis and binding assay suggested that the functional binding of tRNA^Trp^ (the second step of the tRNA aminoacylation reaction) requires the asymmetric conformation of TrpRS and therefore provides possible selection pressure for evolving a half-of-the-sites catalytic mode.

### Fragment screening against the asymmetric state *Ec*TrpRS

The fact that *Ec*TrpRS purified from *E. coli* cells was crystallized as the ‘open-closed’ conformation suggested that this asymmetric state with the intermediate product bound at one of the two active sites might be one of the major states of TrpRS in bacterial cells. Thus, this asymmetric conformation of *Ec*TrpRS provides a valuable new template for discovering TrpRS inhibitors. Fragment screening has been widely used to discover drug leads with novel scaffolds as well as to identify new druggable pockets (40,41). We employed the fluorescence-based thermal shift assay (TSA) to screen a library of 1628 fragments against asymmetric *Ec*TrpRS to identify potential building blocks for TrpRS inhibitors (42). As a control, a parallel fragment screening assay was also performed against apo *Ec*TrpRS (prepared by dialysis). Ligand binding could enhance the melting temperature (*T*_m_) of a protein during its thermal denaturation process, and tighter binders usually cause larger positive shifts of *T*_m_ (34). We considered the fragments (at a final concentration of 1 mM) that caused Δ*T*_m_ (Δ*T*_m_ = *T*_m_(Frg) − *T*_m_(apo)) values of *Ec*TrpRS greater than 1.0°C (approximately threefold of the s.d. of triplicate measurements of the *T*_m_ of *Ec*TrpRS) as positive hits.

**Figure 5.**
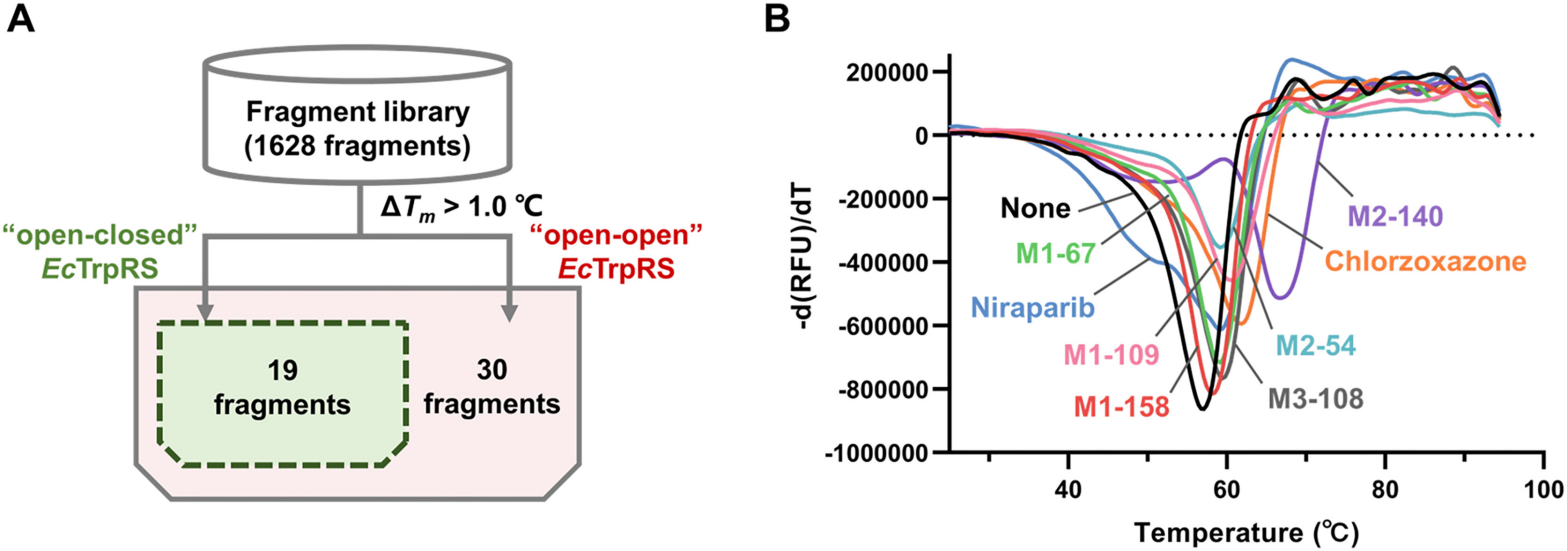
Fragment screening against *Ec*TrpRS using a fluorescence-based thermal shift assay. (**A**) Schematic illustration of the TSA-based fragment screening process. A fragment was identified as a binder of *Ec*TrpRS if it increased the *T*_m_ value of *Ec*TrpRS by > 1.0°C. Fragments were screened in parallel against both the ‘open-open’ *Ec*TrpRS and ‘open-closed’ *Ec*TrpRS. Thirty fragments were identified to bind the ‘open-open’ *Ec*TrpRS, including 19 fragments that could bind the ‘open-closed’ *Ec*TrpRS. (**B**) The representative thermal melting curves of ‘open-closed’ *Ec*TrpRS in the presence of the eight fragments whose binding modes have later been determined by cocrystal structures with *Ec*TrpRS.

Finally, 19 potential hits were identified for the asymmetric *Ec*TrpRS and 30 hits for the apo *Ec*TrpRS (Figure 5A and Supplementary Figure S6). Many of these fragment hits contain a moiety similar to indole, the side chain of substrate L-Trp of *Ec*TrpRS, which supports the reliability of our fragment screening assay. Notably, all 19 hits bound to the asymmetric *Ec*TrpRS are included in the 30 hits for apo *Ec*TrpRS, suggesting that chemicals targeting to the asymmetric bacterial TrpRS have great potential to also bind to apo TrpRS, which would result in more complete inhibition of TrpRS in bacterial cells.

Then, we selected the 19 hits bound to asymmetric *Ec*TrpRS to grow the cocrystals with *Ec*TrpRS. Through efforts, eight cocrystal structures were determined at resolutions between 1.78 and 2.65 Å (Supplementary Figure S5 and Supplementary Table S1). To our surprise, while a fragment named niraparib bound to the L-Trp binding site, the other seven fragments, although three of them contained indole-like aromatic heterocyclic structures, all bound to an unprecedented pocket buried at the interface between the two subunits of *Ec*TrpRS.

### Seven fragments reveal a new binding pocket at the dimeric interface of TrpRS

**Figure 6.**
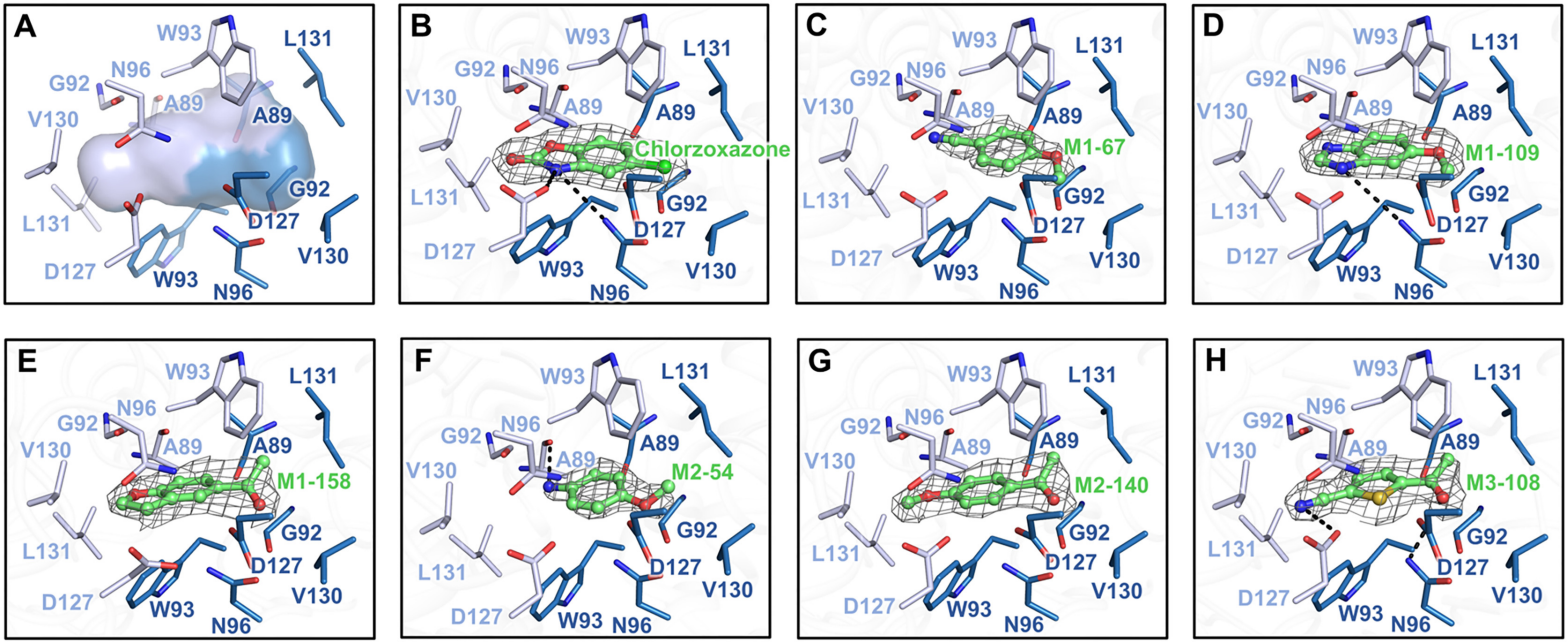
Binding of seven fragments to the dimeric interface of ‘open-closed’ *Ec*TrpRS. (**A**) An unprecedented pocket at the dimeric interface of *Ec*TrpRS. This pocket is built by several hydrophobic and hydrophilic residues (Ala89, Gly92, Trp93, Asn96, Asp127, Val130 and Leu131) from both subunits. Binding of fragments chlorzoxazone (**B**), M1-67 (**C**), M1-109 (**D**), M1-158 (**E**), M2-54 (**F**), M2-140 (**G**) and M3-108 **(H)** to this pocket. Residues from sub^Open^ and sub^Closed^ are colored dark blue and light blue, respectively. The hydrogen bonds are shown as black dashes.

In this study, seven fragments were observed to bind to an unprecedented pocket buried at the dimeric interface between the CP1 domains of two *Ec*TrpRS subunits (Figure 6). This pocket is also observed in bacterial TrpRS in ‘open-open’ and ‘closed-closed’ states (PDB codes: 5V0I and 7ELT), and fragment binding does not significantly change the volume and the shape of this buried pocket, as predicted using DoGSiteScorer (43). The CP1 domains of both subunits contribute equal residues (Ala89, Gly92, Trp93, Asn96, Asp127, Val130 and Leu131) to build this pocket, and this pocket is generally symmetric in shape and shows both hydrophobic and hydrophilic characteristics (Figure 6A). Site-directed mutations were performed on these pocket-surrounding residues, and *Ec*TrpRS variants with D127A, V130A or L131A mutations were successfully expressed and purified. TSA results showed that the fragments caused significantly smaller Δ*T*_m_ values to these *Ec*TrpRS variants than to wild-type *Ec*TrpRS (Supplementary Figure S7), confirming the specific binding of the fragments to this pocket.

Chlorzoxazone is a fragment-like small molecule drug used to relieve pain and stiffness caused by muscle spasms, strains and sprains. The residues Asn96 from sub^Open^ and Asp127 from sub^Closed^ could form hydrogen bonds with the nitrogen of the oxazolone group of chlorzoxazone. In addition, two Trp93 residues from both sub^Open^ and sub^Closed^ contribute hydrophobic contacts and π–π interactions to stabilize chlorzoxazone (Figure 6B). The binding modes of the other six fragments in this pocket are similar to chlorzoxazone, particularly for the hydrophobic interactions contributed by Trp93 from both subunits (Figure 6C–H). Remarkably, although this pocket is generally symmetric in shape, however, all the seven fragments bind to it in an asymmetric way that the electron density maps clearly show a single orientation for each fragment in the pocket. Probably, the binding of TrpAMP caused a slight conformational difference in the CP1 domain between sub^Open^ and sub^Closed^. We used an ITC assay to test the binding of these fragments to *Ec*TrpRS in different states, and the fragment M1-109 gave a detectable heat signal. Consistently, M1-109 showed a slightly better binding affinity to ‘open-closed’ (26.1 ± 4.4 uM) than ‘open-open’ (46.0 ± 10.5 uM) *Ec*TrpRS (Supplementary Figure S8A-B).

The CP1 domain of *Hc*TrpRS adopts a similar structure to *Ec*TrpRS, and a buried pocket also exists at the dimeric interface of *Hc*TrpRS (Supplementary Figure S9A). However, sequence alignments revealed that the CP1 sequences in forming this buried pocket are vary widely between bacterial and eukaryotic TrpRSs (Supplementary Figure S9B). The ITC results showed that M1-109 loses affinity to *Hc*TrpRS (Supplementary Figure S8C, D). Thus, these fragment hits are likely specific to bacterial TrpRS.

The CP1 domain not only serves as a connecting bridge between the two subunits, but also plays crucial roles in forming the L-Trp binding pocket and interacting with the tRNA acceptor arm (22,44). Specially, the important fragment-binding residue Trp93 (numbered as *Ec*TrpRS) is highly conserved in all the aligned bacterial TrpRSs except for *Mycoplasma genitalium* TrpRS in which Trp is replaced by a similar residue Tyr (Supplementary Figure S8). Mutation of Trp93 to Ser resulted in insoluble expression of *Ec*TrpRS, consistent with a previous study on the W93F mutation of *Bacillus subtilis* TrpRS (45). Thus, Trp93 and other fragment-binding residues on the CP1 domain likely play roles in the structure and function of bacterial TrpRS. We then tested whether these fragments could affect the catalytic activity of bacterial TrpRS. However, none of these small fragments could significantly inhibit the activity of *Ec*TrpRS in the tRNA-dependent ATP consumption assay (data not shown). The development of potent drug-like molecules based on these fragments is needed to evaluate the druggability of this pocket.

### The unique and bacterial selective binding mode of niraparib at the active site

**Figure 7.**
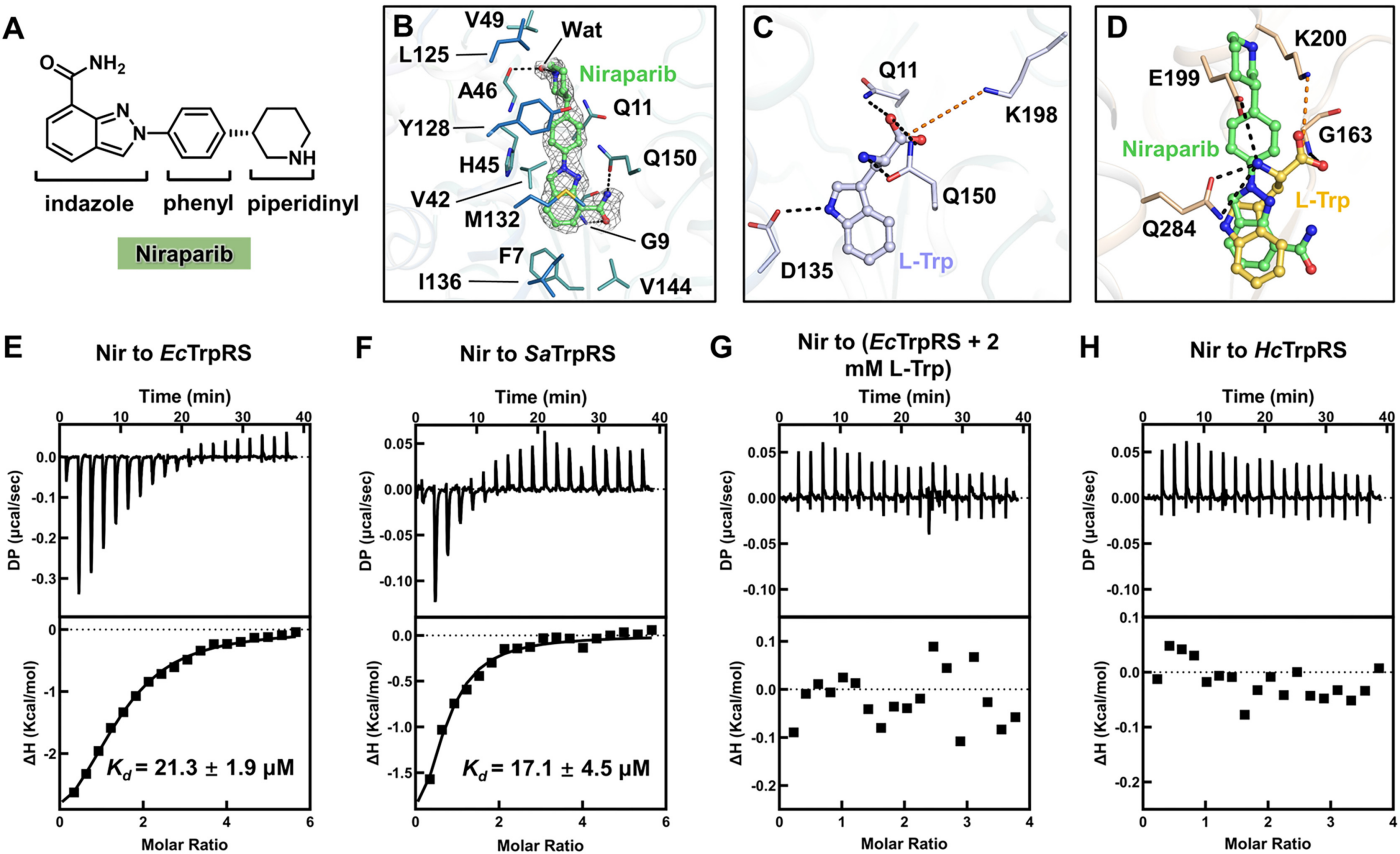
The binding mode and selectivity of niraparib to bacterial TrpRS. (**A**) Chemical structure of niraparib. (**B**) The binding mode of niraparib at the active site of *Ec*TrpRS. An annealed omit electron density map of niraparib calculated with Fourier coefficients 2Fo – Fc and contoured at 1.0 σ. (**C**) The binding mode of L-Trp in *Ec*TrpRS (PDB code 5V0I). (**D**) Structural explanation for the insensitivity of *Hc*TrpRS to niraparib. In (B–D), the hydrogen bonds are shown as black dashes, and salt bridges as yellow dashes. (E–H) ITC experiments showed that niraparib could potently bind to *Ec*TrpRS (**E**) and *Sa*TrpRS (**F**), and this binding was specifically blocked by the high concentration of substrate L-Trp (**G**). In contrast, niraparib did not bind to *Hc*TrpRS (**H**).

Niraparib is a fragment-like small inhibitor against poly ADP-ribose polymerase (PARP), and it is clinically used for treating multiple human cancers including recurrent epithelial ovarian, fallopian tube and primary peritoneal cancer (46). In this study, the cocrystal structure of niraparib bound to the asymmetric *Ec*TrpRS was determined to 2.10 Å resolution (Figure 7A and Supplementary Table S1), and a niraparib molecule was unambiguously modeled into the active site cavity of sub^Open^ according to the electron density map (Figure 7B).

The structure of niraparib could be simply described as linearly linked indazole, phenyl and piperidinyl rings (Figure 7A). The phenyl and piperidinyl rings bind to a cleft formed by residues Gln11, His45, Val49, Leu125 and Tyr128 of *Ec*TrpRS mainly through hydrophobic interactions, and the backbone oxygen of Ala46 forms a water-mediated hydrogen bond with the nitrogen of the piperidinyl ring (Figure 7B). The indazole moiety, a structure similar to the indole group of L-Trp, occupies the L-Trp binding pocket surrounded by hydrophobic residues Phe7, Val42, Met132, Ile136 and Val144. Importantly, the specific recognition of the side chain of L-Trp by *Ec*TrpRS involves a key hydrogen bonding interaction between Asp135 and the nitrogen of the indole moiety (Figure 7C), and this interaction is conserved in the binding of L-Trp to all bacterial TrpRSs (17). Notably, the natural products indolmycin (PDB code: 5DK4) and chuangxinmycin (PDB code: 7CKI) both use an indole-like structure to occupy the L-Trp binding site of bacterial TrpRS, and both form key hydrogen bonding interactions with this conserved aspartate residue, similar to what substrate L-Trp does (20,21). However, this interaction does not exist between niraparib and *Ec*TrpRS. Instead, residues Gly9 and Gln150 of *Ec*TrpRS form three hydrogen bonds with the nitrogen and carboxamide group of niraparib from the opposite side (Figure 7B). Thus, niraparib represents a new mechanism by which chemicals inhibit the L-Trp binding site of bacterial TrpRS, providing a potential way to overcome drug resistance (47).

The key residues for recognizing the indole group of L-Trp in *Hc*TrpRS are different from those in bacterial TrpRS (23). While neither the binding of substrate L-Trp nor inhibitor niraparib causes a significant conformation change to *Ec*TrpRS, the binding of L-Trp to *Hc*TrpRS uses an induced fit mechanism in which the AIDQ motif (corresponding to GEDQ in bacterial TrpRS) moves towards the active site to interact with L-Trp and induces an overall closed conformation of *Hc*TrpRS (23). This closed L-Trp binding pocket of *Hc*TrpRS cannot accommodate indolmycin, which results in an approximately 1000-fold weaker affinity of indolmycin to *Hc*TrpRS than to bacterial TrpRS (20). Here, when niraparib was modeled into the L-Trp binding site of *Hc*TrpRS (PDB code: 2QUH), it clashed with residues Gln284 and Glu199 (Figure 7D). Consistently, niraparib exhibited enzyme inhibitory activity to bacterial TrpRS (IC_50_ = 148 μM) (Supplementary Figure S10), but not *Hc*TrpRS, in the tRNA-denpendent ATP consumption assays, implying that niraparib is unlikely to interfere with protein biosynthesis in human cells through inhibiting *Hc*TrpRS. While the IC_50_ values are associated with the conditions in which the assays have been carried out, we then used the ITC assays to directly measure the binding affinities of niraparib to bacterial TrpRS. The results showed that niraparib has affinities around 20 μM to TrpRSs from both *E. coli* (gram-negative) and *S. aureus* (gram-positive, *Sa*TrpRS) (Figure 7E and F), a value about twofold better than the substrate L-Trp (Figure 2A and B) and better than the affinities of typical fragment hits binding to their targets (40). Notably, this binding of niraparib to bacterial TrpRS is specific to the L-Trp binding site as it could be blocked by adding high concentration of L-Trp (Figure 7G). In contrast, niraparib showed no significant binding to *Hc*TrpRS (Figure 7H). The unique and bacteria-specific binding mode makes niraparib a valuable starting point for developing TrpRS-targeted antibacterials via a fragment-based drug discovery (FBDD) strategy.

## DISCUSSION

### The half-of-the-sites reactivity in bacterial TrpRS and other AARSs

The half-of-the-sites reactivity for homodimeric enzymes refers to a phenomenon in which only one subunit, despite both subunits having the same sequence, is active at a time, and this phenomenon has been frequently observed in many homooligomeric enzymes (48). Most AARSs in class II and TrpRS, TyrRS and MetRS in class I function as homodimers (49). In addition, heterotratetrameric bacterial GlyRS and PheRS are organized as two equal protomers, each containing an active site (50). Previous studies revealed that the two active sites in some oligomeric AARSs, such as TyrRS, LysRS-II, AspRS, HisRS and ProRS (51–55), are not simultaneously active, exhibiting the half-of-the-sites activity similar to that observed in TrpRS. Notably, because crystallization drops contain high concentrations of AARSs and ligands, in most cases, the ligands could be pushed into both active sites, although one of them is less active. For example, TyrRS binds only one molecule of L-Tyr or tRNA^Tyr^ and forms only one molecule of tyrosyl adenylate (TyrAMP) per dimer in solution, showing clear half-of-the-sites activity (56,57). However, TyrRS in crystals forms an artificial symmetric dimer bound with two molecules of L-Tyr, TyrAMP or tRNA^Tyr^ (58,59). Thus, the ‘open-closed’ asymmetric structure of *Ec*TrpRS determined in this study provides a good opportunity for understanding the structural state associated with the half-of-the-sites reactivity.

TrpRS and TyrRS are the only two members of subclass Ic AARSs, and the differentiation between them was suggested occurring at a late stage during the development of genetic codes (17). Impressively, evidences have shown that the active and inactive sites are randomly selected from the two active sites of the TyrRS dimer, and once selected, no interconversion between active and inactive sites could be detected over time (49,51). Thus, the half-of-the-sites reactivity of TyrRS is likely caused by its preexisting inherent asymmetry. The underlying mechanism for TrpRS is not such clear. The asymmetric binding of 7 fragments at the dimer interface supports a potential preexisting asymmetry for *Ec*TrpRS (Figure 6), but the unfavorable binding of the second ATP but not L-Trp (Figure 2) suggested that it might be the strong negative cooperation between two ATP binding sites causes the half-of-the-sites reactivity in bacterial TrpRS. Interestingly, regardless of how bacterial TrpRS achieves the half-of-the-sites reactivity, our structural analysis and binding assays suggested that the ‘open-closed’ asymmetric conformation is necessary for the functional binding of tRNA^Trp^ (Figure 3), which may provide the evolutionary pressure for driving the formation of the half-of-the-sites reactivity in bacterial TrpRS.

### Implications of dimer stabilization effects of the fragments buried at the interface

In this study, seven fragments were observed to enter the pocket buried at the dimeric interface of *Ec*TrpRS, suggesting a monomer-dimer exchange of *Ec*TrpRS in solution. Once *Ec*TrpRS in different states was diluted to 3.5 μM and loaded onto gel-filtration column, both ‘open-closed’ and ‘closed-closed’ *Ec*TrpRSs (bound with one or two TrpAMP molecules respectively) dominantly existed as dimers, but apo *Ec*TrpRS formed almost equal dimer and monomer peaks (Supplementary Figure S11). Incubation with 200 μM chlorzoxazone could increase the dimer peak and decrease the monomer peak for apo *Ec*TrpRS, suggesting that binding of these fragments to the dimer interface did not disrupt but actually facilitated stabilization of the *Ec*TrpRS dimer. Considering that dimerization is necessary for the cross-subunit binding of tRNA^Trp^ to TrpRS (39), these fragments may help to maintain the activity of bacterial TrpRS when the TrpRS concentration is low *in vivo*.

Charcot-Marie-Tooth disease (CMT) is one of the most common inherited neuropathies without available clinical treatment. AARSs are the largest known protein family associated with the etiology of CMT, and mutations in seven human cytoplasmic AARSs including TrpRS have been reported to cause CMT via either the loss-of-function mechanism (e.g. mutations weaken the tRNA charging activity of AARSs) or the gain-of-function mechanism (e.g. gain of new protein-protein interactions toxic to neuron cells) (60,61). Consequently, different therapeutic strategies have been proposed. Overexpression of the corresponding substrate tRNA was shown to rescue protein synthesis levels and attenuate CMT caused by AARS mutations (62). For the second mechanism, an example is that overexpression of vascular endothelial growth factor (VEGF, a natural ligand of the axon guidance receptor neuropilin1) could compete off the pathogenic interaction between neuropilin1 and mutant GlyRS and rescue the CMT phenotype in a mouse model (63). Notably, it has been proven that most CMT mutations of AARSs are related to the weakened dimer (61,64). Dimer disassociation likely plays an important role in both the loss-of-function and gain-of-function mechanisms of the development of AARS-associated CMT diseases (61,65). In this study, gel filtration assays revealed that even wild-type *Ec*TrpRS has the potential to dissociate from dimer to monomer, especially in the apo state. Fragment screening and crystallography identified seven chemical fragments that are buried between two subunits of *Ec*TrpRS. Importantly, insertion of these chemicals at the dimer interface did not disrupt the dimerization nor impair enzyme activity (at least for these small fragments), but actually helped to stabilize the dimer according to gel filtration assays (Supplementary Figure S11). Probably, searching for small molecules that stabilize human AARS dimers [e.g. molecules binding to the buried pocket at the dimeric interface of *Hc*TrpRS (Supplementary Figure S8A)] could be an alternative strategy for developing drugs to treat related human diseases.

In conclusion, this study determined an ‘open-closed’ *Ec*TrpRS structure with a copurified TrpAMP bound in sub^Closed^, providing not only direct structural evidence for the long-discussed half-of-the-sites reactivity of bacterial TrpRS but also a possible reason why bacterial TrpRS requires this half-of-the-sites reactivity. Fragment screening identified 19 hits against ‘open-closed’ asymmetric *Ec*TrpRS. With eight cocrystal structures, we clarified the unique binding mode of niraparib at the active site, and also identified an unprecedented pocket at the dimeric interface of bacterial TrpRS. These findings improve the understanding of the structural and catalytic mechanism of bacterial TrpRS and will help AARS-based antimicrobial discovery in the future.

## DATA AVAILABILITY

Atomic coordinates and structure factors for the reported crystal structures have been deposited with the Protein Data Bank under accession numbers 8I1W, 8I4I, 8I27, 8I2A, 8I1Z, 8I2C, 8I2J, 8I1Y, 8I2L and 8I2M.

## Supplementary Material

gkad278_Supplemental_File
