# Supplementary material for: An asymmetric structure of bacterial TrpRS supports the half-of-the-sites catalytic mechanism and facilitates antimicrobial screening
Source: Nucleic Acids Res. 2023 Apr 18;51(9):4637–49. doi: 10.1093/nar/gkad278 (PMC10201369; doi:10.1093/nar/gkad278)
Supplement: gkad278_Supplemental_File [file gkad278_supplemental_file.pdf]

Supplementary Information

An asymmetric structure of bacterial TrpRS supports the half-of-the-sites catalytic mechanism and facilitates antimicrobial screening

Manli Xiang, Kaijiang Xia, Bingyi Chen, Zhiteng Luo, Ying Yu, Lili Jiang, Huihao Zhou*

Guangdong Provincial Key Laboratory of Chiral Molecule and Drug Discovery and Research Center for Drug Discovery, School of Pharmaceutical Sciences, Sun Yat-sen University, Guangzhou 510006, China

* To whom correspondence should be addressed. Tel: +86 20 39943350; Email: zhuihao@mail.sysu.edu.cn

Supplementary Figures

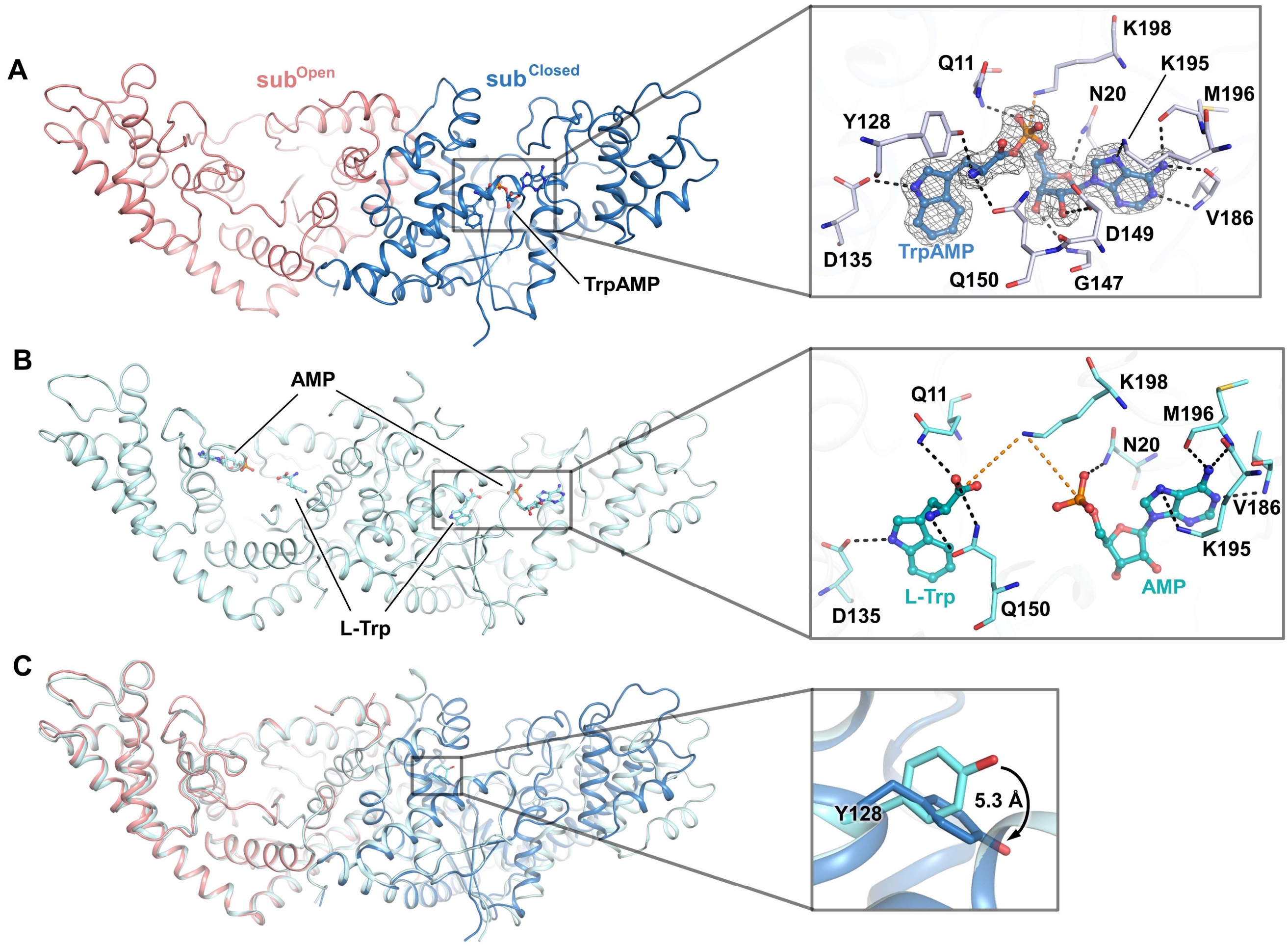

Supplementary Figure S1. The overall structures of *Ec*TrpRS in different states. (A) The structure of *Ec*TrpRS in complex with a molecule of TrpAMP (the sub^{Open} is colored in salmon, and the sub^{Closed} in skyblue). The $2F_o$ - F_c omit electron density map around TrpAMP is drawn as grey meshes contoured at 1.0 σ. **(B)** The structure of *Ec*TrpRS in complex with L-Trp and AMP (palecyan, PDB code 5V0I). **(C)** Structural superposition of the *Ec*TrpRS·TrpAMP complex to the *Ec*TrpRS·L-Trp·AMP complex by aligning the ADs of *Ec*TrpRS. The side chain of Tyr128 rotates downwards (~ 5.3 Å for its phenolic hydroxyl) to interact with the α-amino group of the L-Trp moiety of TrpAMP. Hydrogen bonds are shown as black dashes, while salt bridges are shown as yellow dashes.

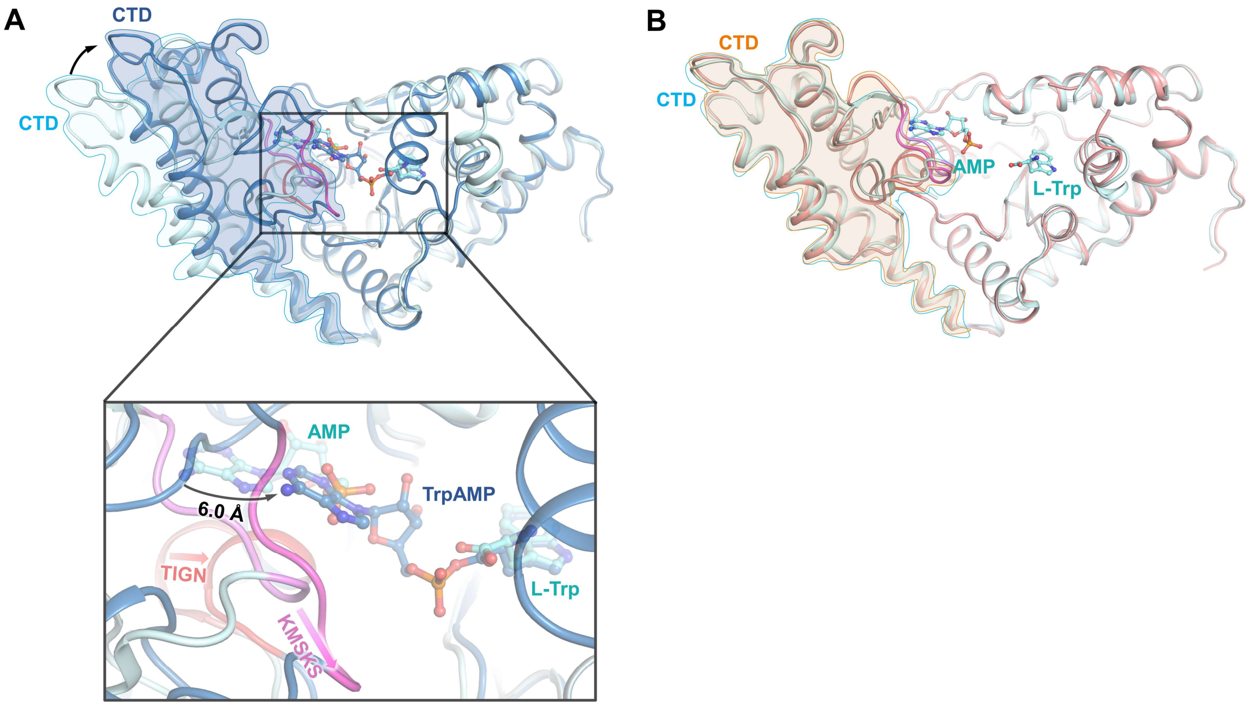

Supplementary Figure S2. Structural comparison of *Ec*TrpRS subunits bound with or without ligands. (A) Superposition of the *Ec*TrpRS subunit bound with TrpAMP (sub^{Closed}, skyblue) to the *Ec*TrpRS subunit bound with L-Trp and AMP (palecyan, PDB code 5V0I) by aligning their aminoacylation domains (ADs). Compared to the AMP molecule, the AMP portion of TrpAMP moves approximately 6.0 Å to attach to the L-Trp moiety, which couples with conformation changes of the class I signature motifs (TIGN and KMSKS) and CTD. **(B)** Superposition of the *Ec*TrpRS subunit in apo form (sub^{Open}, salmon) to the *Ec*TrpRS subunit in complex with L-Trp and AMP by aligning their ADs. Two *Ec*TrpRS subunits show a similar conformation. The TIGN and KMSKS motifs are colored in magenta. The CTDs of sub^{Closed}, sub^{Open} and the *Ec*TrpRS subunit bound with L-Trp and AMP are highlighted in sky blue, salmon and pale cyan, respectively.

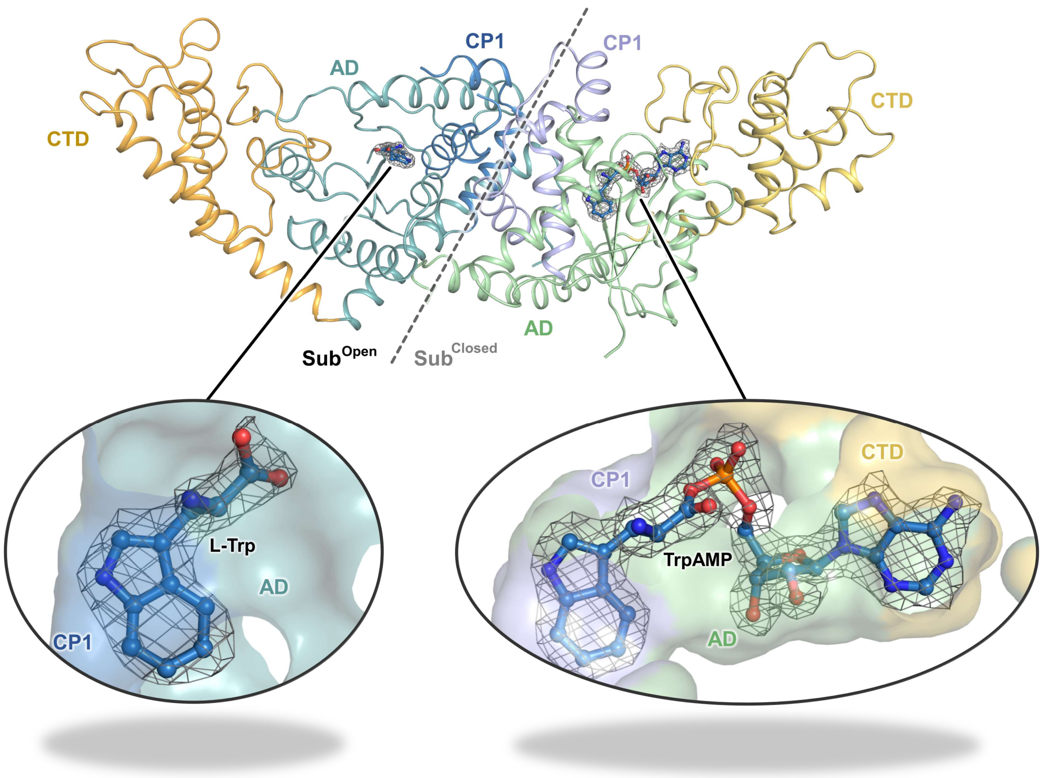

Supplementary Figure S3. The structure of *Ec*TrpRS bound with L-Trp and TrpAMP. An overview of the *Ec*TrpRS·TrpAMP·L-Trp complex structure was determined at a resolution of 2.20 Å. 2F_o - F_c omit electron density maps for L-Trp and TrpAMP are shown as grey meshes contoured at 1.0 σ.

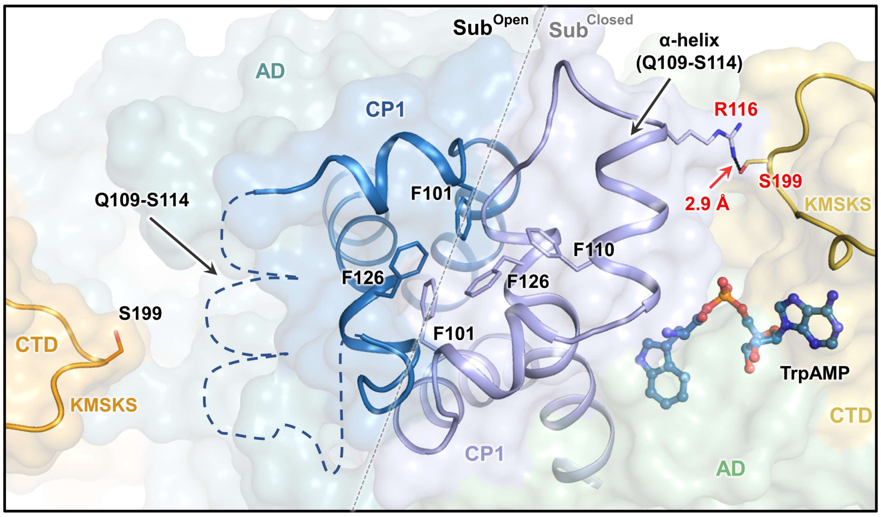

Supplementary Figure S4. A possible way to bridge two ATP binding sites of *Ec*TrpRS. The short α-helix (Q109-S114) in the CP1 domain may contribute to stabilizing (e.g., through the hydrogen bond interaction between R116 and S199) the conformation of the KMSKS loop, a class I AARSs signature motif important for ATP binding. Two CP1 domains interact with each other by forming a unified hydrophobic interaction network, which may serve as a bridge to connect two ATP binding sites. The hydrogen bond is shown as a black dashed line.

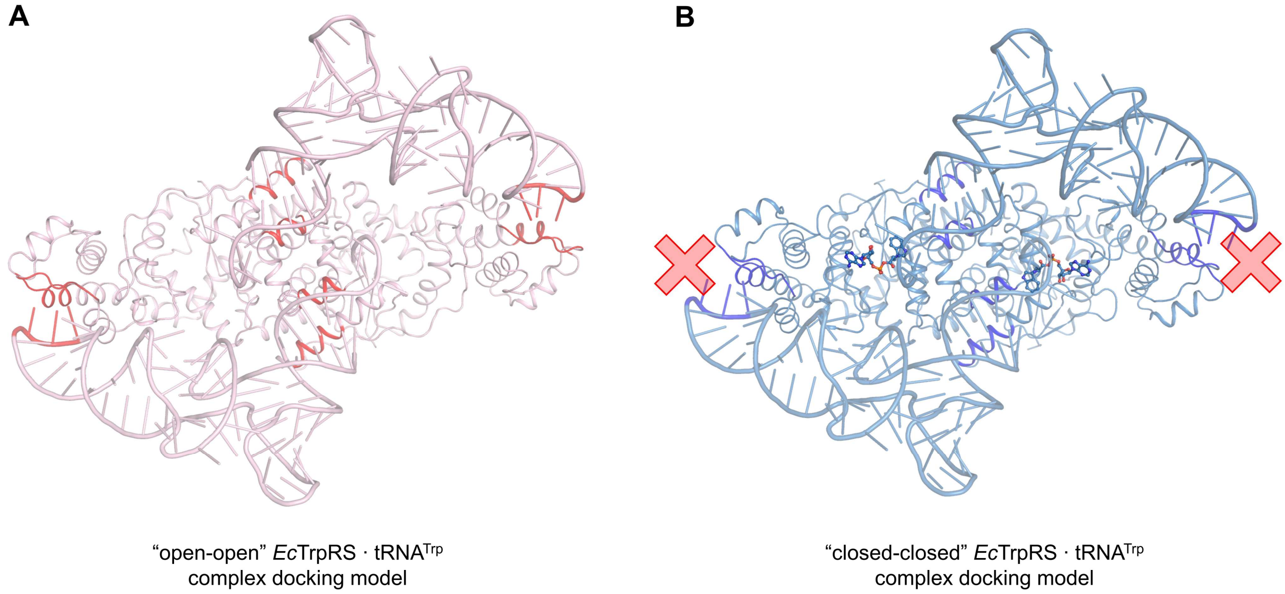

Supplementary Figure S5. The docking models of the *Ec*TrpRS·tRNA^{Trp} complex. (A) The docking model of the "open–open" *Ec*TrpRS·tRNA^{Trp} complex. **(B)** The docking model of the "closed–closed" *Ec*TrpRS·tRNA^{Trp} complex suggests mismatches between the anticodons of tRNA^{Trp} and the anticodon binding sites of *Ec*TrpRS.

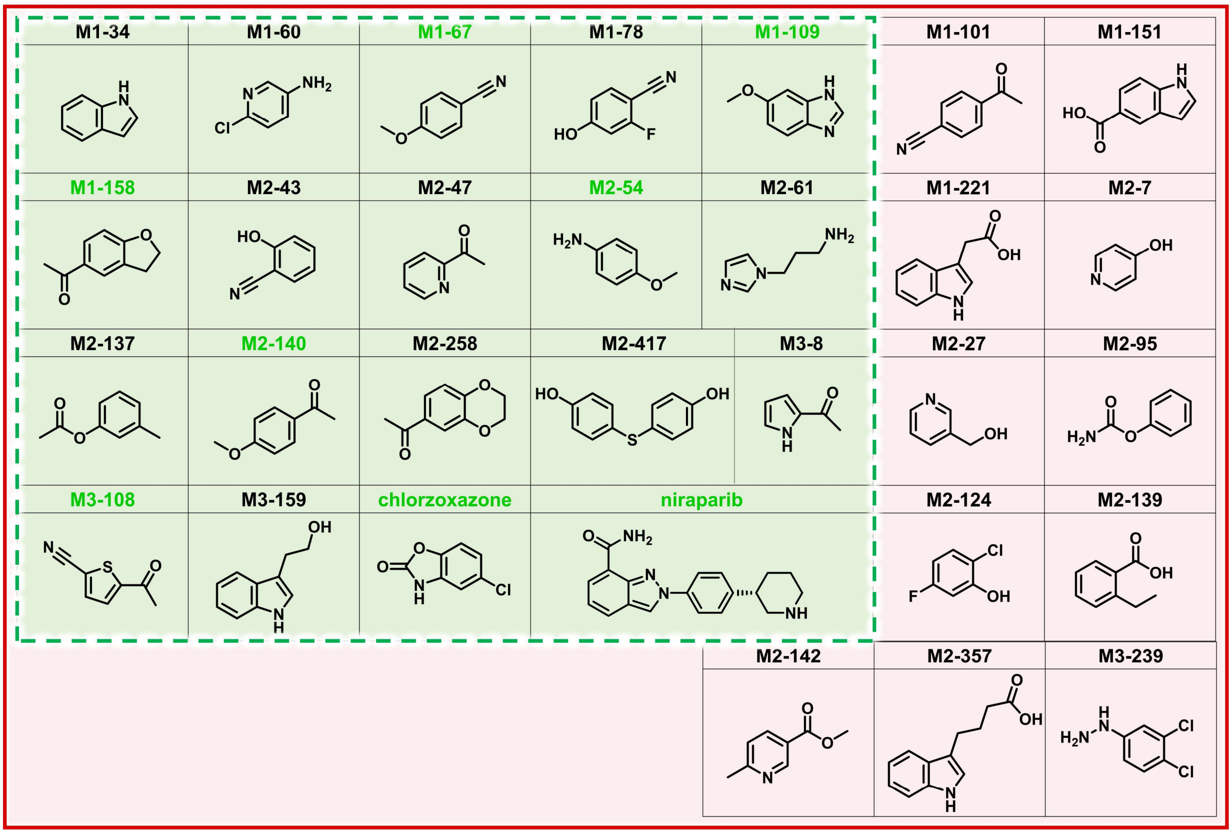

Supplementary Figure S6. The chemical structures of *Ec*TrpRS binding fragments identified by fluorescence-based thermal shift assay. Nineteen fragments that could increase the T_m value of the "open–closed" *Ec*TrpRS by >1.0 °C were identified as hits (listed in the green box). All nineteen fragments were included in the thirty fragments that could increase the T_m value of the "open–open" *Ec*TrpRS by >1.0 °C (listed in the red box). The numbers/names of the eight fragments which were determined the cocrystal structures with "open–closed" *Ec*TrpRS are colored green.

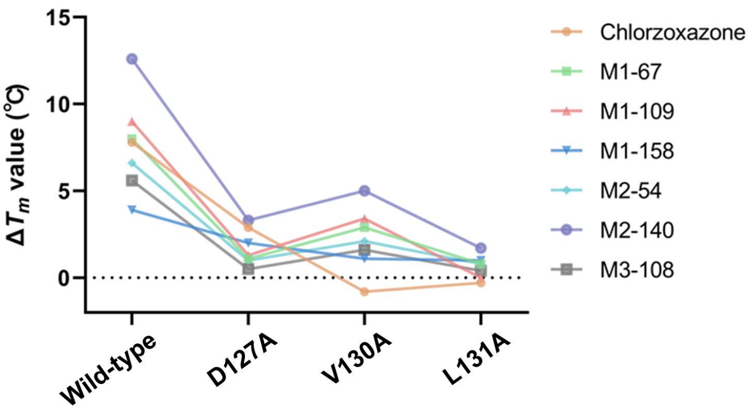

Supplementary Figure S7. Mutations of pocket-forming residues disrupted the binding of fragments to *Ec*TrpRS as tested by TSA. The D127A, V130A and L131A mutations largely reduced the ΔT_m values of *Ec*TrpRS caused by the fragments.

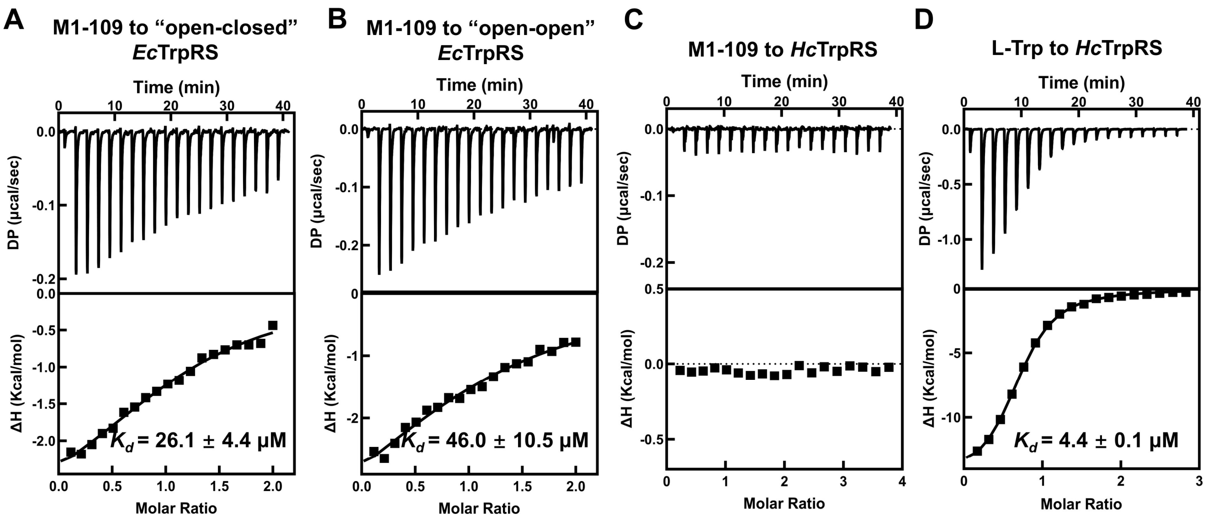

Supplementary Figure S8. The binding of the fragment M1-109 to TrpRS. (A, B) ITC assay revealed that fragment M1-109 has slightly better binding affinity to “open–closed” *Ec*TrpRS than to “open–open” *Ec*TrpRS. **(C, D)** M1-109 did not show significant binding to *Hc*TrpRS, although the correct folding of *Hc*TrpRS overexpressed in *E. coli* has been confirmed by its potent binding with substrate L-Trp.

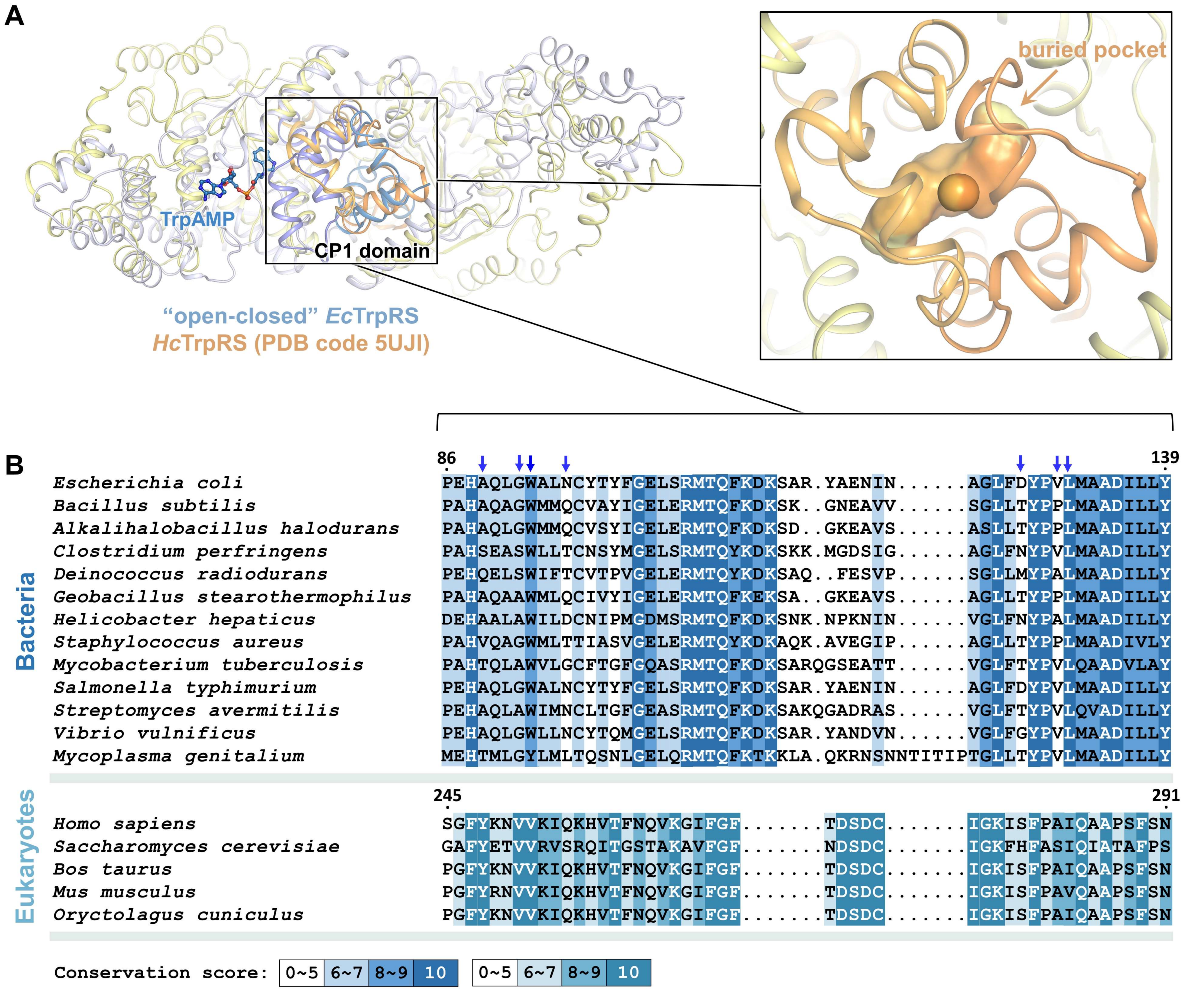

Supplementary Figure S9. Structural and sequence comparisons between the CP1 domains of bacterial and eukaryotic TrpRSs. (A) Superposition of *Ec*TrpRS·TrpAMP and *Hc*TrpRS (PDB code 5UJI) by aligning their ADs. The buried pocket existing at the dimeric interface of *Hc*TrpRS is drawn as the surface. **(B)** The sequences of CP1 domains of TrpRSs from *E. coli* (UniprotKB ID P00954), *Bacillus subtilis* (UniprotKB ID P21656), *Alkalihalobacillus halodurans* (UniprotKB ID Q9K8Y2), *Clostridium perfringens* (UniprotKB ID Q8XMQ5), *Deinococcus radiodurans* (UniprotKB ID Q9RWV7), *Geobacillus stearothermophilus* (UniprotKB ID P00953), *Helicobacter hepaticus* (UniprotKB ID Q7VIP6), *S. aureus* (UniprotKB ID Q6GI89), *Mycobacterium tuberculosis* (UniprotKB ID P9WFT2), *Salmonella typhimurium* (UniprotKB ID P0A2P2), *Streptomyces avermitilis* (UniprotKB ID Q82HU1), *Vibrio vulnificus* (UniprotKB ID Q7MH15), *Mycoplasma genitalium* (UniprotKB ID P47372), *Homo sapiens* (UniprotKB ID P23381), *Saccharomyces cerevisiae* (UniprotKB ID Q12109), *Bos taurus* (UniprotKB ID P17248), *Mus musculus* (UniprotKB ID P32921) and *Oryctolagus cuniculus* (UniprotKB ID P23612) were aligned using the program MAFFT with manually adjustments (1). Residues are colored according to the conservation score calculated by the program PRALINE (2). Residues contributing to building the pocket located at the dimeric interface are labeled by arrows.

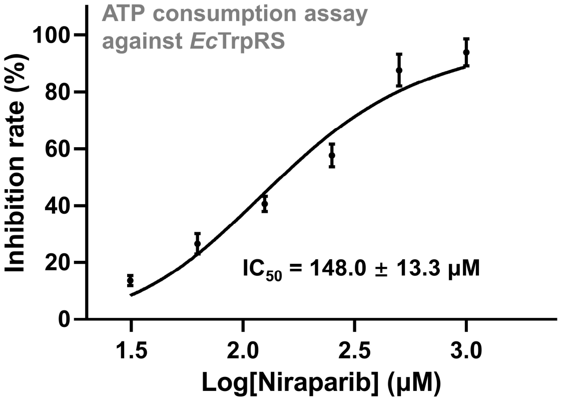

Supplementary Figure S10. The inhibitory activity of niraparib against *Ec*TrpRS. Niraparib inhibition of the catalytic activity of *Ec*TrpRS in the tRNA-dependent ATP consumption assay.

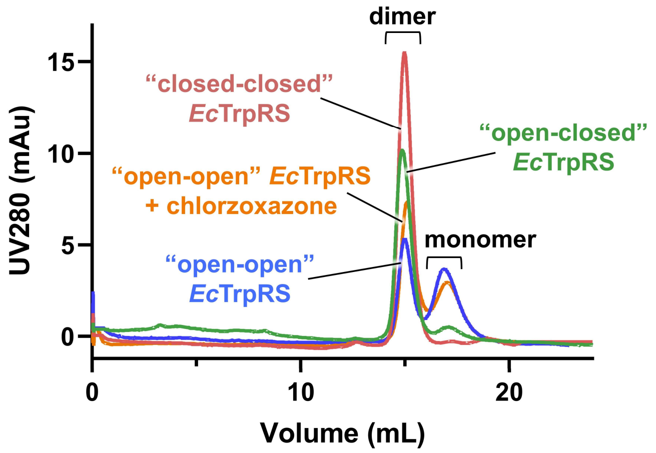

Supplementary Figure S11. The elution profile of TrpRS at different states in size exclusion chromatography. While the “open–closed” and “closed–closed” *Ec*TrpRSs dominantly formed a single peak of homodimer, the “open–open” *Ec*TrpRS existed as both homodimer and monomer in solution. Incubation with 200 μM chlorzoxazone increased the dimer peak and reduced the monomer peak, suggesting that the fragment bound to the buried pocket at the dimeric interface could help to stabilize the dimerization of bacterial TrpRS.

Supplementary Table

Table S1. Data collection and refinement statistics.

(Table S1, Part I)

	*Ec*TrpRS · TrpAMP	*Ec*TrpRS · TrpAMP · L-Trp	*Ec*TrpRS · TrpAMP · M1-67	*Ec*TrpRS · TrpAMP · M1-109
**PDB code**	8I1W	8I4I	8I27	8I2A
**Data collection**				
X-ray source	SSRF BL02U1	Rigaku MicroMax-007 HF	SSRF BL19U1	SSRF BL19U1
Wavelength (Å)	0.9792	1.5418	0.9785	0.9785
Resolution (Å)	50.00-1.80(1.84-1.80) [a]	50.00-2.20(2.32-2.20)	50.00-1.95(2.00-1.95)	50.00-2.35(2.44-2.35)
Space group	$P2_1$	$P2_1$	$P2_1$	$P2_1$
Cell dimensions				
a, *b*, *c* (Å)	61.68, 79.68, 77.65	61.66, 80.05, 77.43	61.72, 80.59, 77.16	61.74, 80.54, 77.26
α, *β*, *γ* (°)	90.00, 106.04, 90.00	90.00, 106.20, 90.00	90.00, 106.22, 90.00	90.00, 106.30, 90.00
Unique reflections	65978(3863)	36816(5332)	51416(3416)	30223(3155)
R_{merge} [b]	0.05(0.44)	0.12(0.48)	0.05(0.40)	0.11(0.54)
Average $I/\sigma(I)$	18.2(4.6)	12.7(3.4)	12.9(2.8)	7.0(2.0)
Completeness (%)	98.8(98.3)	99.9(100.0)	97.3(91.8)	99.5(99.6)
Redundancy	6.5(6.9)	5.4(3.8)	3.4(3.1)	3.4(3.3)
**Refinement**				
Resolution (Å)	50.00-1.80	50.00-2.20	50.00-1.95	50.00-2.35
No. reflections	62587	34885	48799	28710
R_{work} [c]/R_{free} [d] (%)	18.6(22.2)	20.7(22.9)	19.1(22.6)	21.4(24.4)
No. non-hydrogen atoms				
Protein	5102	5015	5041	5011
Ligand/ion	57	62	56	62
Water oxygen atoms	429	246	272	119
Mean B factor (Å^2)	30.3	21.0	29.3	35.6
RMSD bond (Å)	0.006	0.006	0.007	0.007
RMSD angle (°)	1.15	1.11	1.16	1.10
Ramachandran plot (%)				
Favored	97.0	96.6	96.9	97.4
Allowed	2.6	3.2	2.9	2.3
Outliers	0.3	0.2	0.2	0.3

(Table S1, Part II)

	*Ec*TrpRS · TrpAMP · M1-158	*Ec*TrpRS · TrpAMP · M2-54	*Ec*TrpRS · TrpAMP · M2-140	*Ec*TrpRS · TrpAMP · M3-108
**PDB code**	8I1Z	8I2C	8I2J	8I1Y
**Data collection**				
X-ray source	SSRF BL02U1	SSRF BL19U1	Rigaku MicroMax-007 HF	SSRF BL02U1
Wavelength (Å)	0.9792	0.9785	1.5418	0.9792
Resolution (Å)	50.00-1.80(1.84-1.80)	50.00-2.07(2.13-2.07)	50.00-2.80(2.79-2.65)	50.00-1.78(1.82-1.78)
Space group	$P2_1$	$P2_1$	$P2_1$	$P2_1$
Cell dimensions				
a, *b*, *c* (Å)	61.79, 80.15, 78.70	61.72, 80.43, 77.14	61.80, 80.42, 78.66	61.69, 79.97, 78.54
α, *β*, *γ* (°)	90.00, 105.77, 90.00	90.00, 106.05, 90.00	90.00, 105.65, 90.00	90.00, 105.79, 90.00
Unique reflections	67909(4029)	44074(3433)	21631(3166)	70259(3940)
R_{merge}^{b}	0.04(0.33)	0.06(0.49)	0.14(0.41)	0.04(0.53)
Average $I/\sigma(I)$	11.6(2.2)	13.5(2.3)	9.6(3.6)	18.4(2.2)
Completeness (%)	99.2(98.7)	99.5(99.5)	99.7(100.0)	99.8(98.6)
Redundancy	3.0(2.2)	3.4(3.5)	4.0(4.1)	6.0(4.1)
**Refinement**				
Resolution (Å)	50.00-1.80	50.00-2.07	50.00-2.65	50.00-1.78
No. reflections	64433	41849	20518	66833
$R_{work}^{c}/R_{free}^{d}$ (%)	19.5(22.9)	20.1(23.8)	22.5(26.0)	19.2(22.4)
No. non-hydrogen atoms				
Protein	5109	5043	5004	5087
Ligand/ion	64	55	53	72
Water oxygen atoms	361	213	72	486
Mean B factor ($Å^2$)	34.2	35.0	21.6	29.4
RMSD bond (Å)	0.007	0.007	0.007	0.004
RMSD angle (°)	1.16	1.22	1.05	1.22
Ramachandran plot (%)				
Favored	97.1	97.1	96.7	96.8
Allowed	2.8	2.6	3.1	3.2
Outliers	0.2	0.3	0.2	0.0

(Table S1, Part III)

	*Ec*TrpRS · TrpAMP· Chl	*Ec*TrpRS · TrpAMP · Nir
**PDB code**	8I2L	8I2M
**Data collection**		
X-ray source	SSRF BL19U1	Rigaku MicroMax-007 HF
Wavelength (Å)	0.9785	1.5418
Resolution (Å)	50.00-1.95(2.00-1.95)	50.00-2.10(2.21-2.10)
Space group	$P2_1$	$P2_1$
Cell dimensions		
a, *b*, *c* (Å)	61.62, 80.05, 78.28	61.66, 79.76, 77.34
α, *β*, *γ* (°)	90.00, 105.69, 90.00	90.00, 106.32, 90.00
Unique reflections	53051(3657)	42029(6115)
R_{merge}[b]	0.06(0.32)	0.12(0.57)
Average *I/σ(I)*	10.9(2.6)	11.1(2.5)
Completeness (%)	99.4(97.5)	99.9(100.0)
Redundancy	3.3(2.9)	5.0(3.2)
**Refinement**		
Resolution (Å)	50.00-1.95	50.00-2.10
No. reflections	50390	39904
R_{work}[c]/R_{free}[d] (%)	19.9(22.2)	20.2(23.5)
No. non-hydrogen atoms		
Protein	5079	5049
Ligand/ion	57	76
Water oxygen atoms	318	349
Mean B factor (Å^2)	33.8	20.3
RMSD bond (Å)	0.007	0.007
RMSD angle (°)	1.20	1.16
Ramachandran plot (%)		
Favored	97.1	96.8
Allowed	2.8	2.9
Outliers	0.2	0.3

[a] Values in parentheses are for the highest resolution shell.

[b] $R_{merge} = \sum_h \sum_l |I(h)_l - \langle I(h)\rangle| / \sum_h \sum_l I(h)_l$, where $I(h)_l$ is the *l*th observation of the reflection h and $\langle I(h)\rangle$ is the weighted average intensity for all observations *l* of reflection h.

[c] $R_{work} = \sum h||F_{obs}(h)| - |F_{cal}(h)||/\sum_h |F_{obs}(h)|$, where $F_{obs}(h)$ and $F_{cal}(h)$ are the observed and calculated structure factors for reflection h respectively.

[d] R_{free} was calculate as R_{work} using 5% of the reflections which were selected randomly and omitted from refinement.

Supplementary References

1. Katoh, K., Misawa, K., Kuma, K. and Miyata, T. (2002) MAFFT: a novel method for rapid multiple sequence alignment based on fast Fourier transform. *Nucleic Acids Res.*, **30**, 3059-3066.
2. Heringa, J. (2002) Local weighting schemes for protein multiple sequence alignment. *Comput. Chem.*, **26**, 459-477.
